# Red blood cell thickness is evolutionarily constrained by slow, hemoglobin-restricted diffusion in cytoplasm

**DOI:** 10.1038/srep36018

**Published:** 2016-10-25

**Authors:** Sarah L. Richardson, Pawel Swietach

**Affiliations:** 1 Department of Physiology, Anatomy and Genetics, Oxford OX1 3PT, European Union, United Kingdom

## Abstract

During capillary transit, red blood cells (RBCs) must exchange large quantities of CO_2_ and O_2_ in typically less than one second, but the degree to which this is rate-limited by diffusion through cytoplasm is not known. Gas diffusivity is intuitively assumed to be fast and this would imply that the intracellular path-length, defined by RBC shape, is not a factor that could meaningfully compromise physiology. Here, we evaluated CO_2_ diffusivity (D_CO2_) in RBCs and related our results to cell shape. D_CO2_ inside RBCs was determined by fluorescence imaging of [H^+^] dynamics in cells under superfusion. This method is based on the principle that H^+^ diffusion is facilitated by CO_2_/HCO_3_^−^ buffer and thus provides a read-out of D_CO2_. By imaging the spread of H^+^ ions from a photochemically-activated source (6-nitroveratraldehyde), D_CO2_ in human RBCs was calculated to be only 5% of the rate in water. Measurements on RBCs containing different hemoglobin concentrations demonstrated a halving of D_CO2_ with every 75 g/L increase in mean corpuscular hemoglobin concentration (MCHC). Thus, to compensate for highly-restricted cytoplasmic diffusion, RBC thickness must be reduced as appropriate for its MCHC. This can explain the inverse relationship between MCHC and RBC thickness determined from >250 animal species.

The principal biological function of red blood cells (RBCs) is to exchange large volumes of O_2_ and CO_2_ during their brief (typically <1 s) transit through the microvasculature^1^. The speed of gas turnover by RBCs is therefore a measure of their physiological fitness, and highly-conserved biological adaptations are expected to relate to faster gas exchange. The steps in the gas exchange cascade include gas permeation across the cell membrane and binding onto hemoglobin. Additionally in the case of CO_2_, gas molecules are converted reversibly to HCO_3_^−^ ions (which are then transported by anion exchanger 1, AE1) plus H^+^ ions (which are buffered by hemoglobin). These processes are coupled together by cytoplasmic diffusion. According to the prevailing consensus, efficiency of gas exchange is strongly dependent on protein-facilitated membrane transport, including AE1-assisted HCO_3_^−^ transport and aquaporin1-assisted gas permeation^2^,^3^.

A conserved and characteristic feature of human and animal RBCs is their flattened shape. The physiological relevance of this geometry has largely been attributed to the mechanical benefits it offers to circulating blood. RBC flattening allows microvasculature to co-evolve smaller luminal diameters and therefore maximize capillary density. In the case of human RBCs, the biconcave shape supports laminar flow and shear-thinning^4^, minimizes platelet scatter and atherogenic risk^5^, permits cells to squeeze through microvasculature^6^,^7^ and is the lowest energy-level that the cell returns to following deformation in capillaries^8^,^9^,^10^,^11^. Elliptical or otherwise flattened RBCs of many non-human species may also manifest, to some degree, these mechanical benefits. Recently, cell geometry has been proposed as a stringent criterion by which splenic inter-endothelial slits select healthy RBCs for continued circulation^12^, but it is unclear if similar criteria apply in non-human species. These aforementioned mechanical considerations do not, however, offer a unifying explanation for the wide range of RBC thicknesses observed naturally in different animal species, and its inverse relationship with mean corpuscular hemoglobin concentration (MCHC)^13^,^14^,^15^. We postulate that RBC thickness and its relationship with MCHC may, instead, relate more closely to the efficiency of gas turnover.

Theoretically, the flattened RBC form may facilitate gas exchange in two ways. Firstly, it increases the cell’s surface area/volume ratio (ρ), which allows faster membrane transport. Secondly, it collapses the path-length for cytoplasmic diffusion, which reduces the time delays associated with intracellular gas transport. In the case of human RBCs (major radius, r, of 4 μm), adapting the shape from a hypothetical sphere (volume 268 fl; surface area 200 μm^2^) to a flattened form (volume 90 fl; surface area 136 μm^2^) doubles ρ and hence transmembrane flux^13^,^14^,^15^. The significance of this effect can be evaluated by considering the slowest membrane transport process, AE1-facilitated HCO_3_^−^ transport, which has an apparent permeability constant^3^ (P_m,HCO3_) of 18 μm/s and can be ascribed a time constant equal to 1/(ρ × P_m,HCO3_). The AE1-related time constant for the spherical geometry is 0.074 s, which is reduced to 0.037 s for the flattened shape, yet both are compatible with equilibration during capillary transit. The biconcave shape of human RBCs reduces the mean cytoplasmic path-length to 0.9 μm (equal to the cell’s average half-thickness, h), which shortens intracellular diffusion time delays by a factor of seven, compared to a spherical RBC variant (r^2^/6 ÷ h^2^/2). However, this seven-fold acceleration will not translate into a meaningful improvement to gas exchange efficiency if gas diffusion coefficients are high, as they are in water. Paradoxically, gas diffusivity inside gas-carrying RBCs has not been measured, but is intuitively assumed to be rapid. Indeed, O_2_ and CO_2_ diffusivity measurements in hemoglobin solutions and hemolysates (~10^3^ μm^2^/s) appear to support this assertion^2^,^14^,^16^. At this magnitude of diffusivity, cytoplasmic path-length and cell shape are *not* expected to be critical for determining the efficiency of gas exchange. In summary, our current understanding of RBC physiology predicts only a modest advantage of the flattened RBC shape for gas exchange, but this inference is based on an indirect characterization of gas transport inside the RBC.

In this study, we revisited the notion that gases diffuse rapidly in RBC cytoplasm. The required experimental approach must be capable of resolving diffusion on the scale of a single intact cell, and exclude contributions from permeation across extracellular unstirred layers and the surface membrane. To meet these criteria, we evaluated CO_2_ diffusivity (D_CO2_) in intact RBCs by measuring the ability of CO_2_/HCO_3_^−^ to facilitate cytoplasmic H^+^ diffusion^17^,^18^,^19^. This measurement method is based on the observation that cytoplasmic H^+^ ions are heavily buffered^20^, and therefore diffuse only as fast as the buffers there are bound to (with essentially no free ion movement)^21^,^22^. Thus, by determining the CO_2_/HCO_3_^−^-dependent component of buffer-facilitated H^+^ diffusion^17^,^18^,^23^, it is possible to quantify D_CO2_. Our measurements on human RBCs demonstrate substantially restricted CO_2_ diffusivity, to 5% of the rate in water, which is an order of magnitude slower than estimates made previously in cell-free hemoglobin solutions. By imaging osmotically-swollen human RBCs (to dilute MCHC) and RBCs from species with different hemoglobin concentrations, we demonstrate that D_CO2_ decreases sharply with MCHC, consistent with hemoglobin-imposed tortuosity to small-molecule diffusion. We conclude that diffusion across cytoplasm is a hitherto unrecognized rate-limiting step for gas exchange which imposes a critical limit on the RBC thickness, beyond which physiological function becomes inefficient and incomplete. In particular, the full manifestation of the Bohr Effect^24^ (i.e. the process by which hemoglobin releases O_2_ at acidic vascular beds) is attainable only in RBCs that are adequately thin because the underlying H^+^ trigger is transmitted intracellularly by slow CO_2_/HCO_3_^−^ diffusion. Highly restricted cytoplasmic diffusivity can explain the inverse RBC thickness/MCHC relationship observed amongst different animal species, and highlights the potential vulnerability of gas exchange efficiency in diseases that involve a change in RBC shape, such as in spherocytosis.

## Results

### Using measurements of spatio-temporal [H^+^] dynamics as a read-out of CO_2_ diffusion

**Figure 1:**
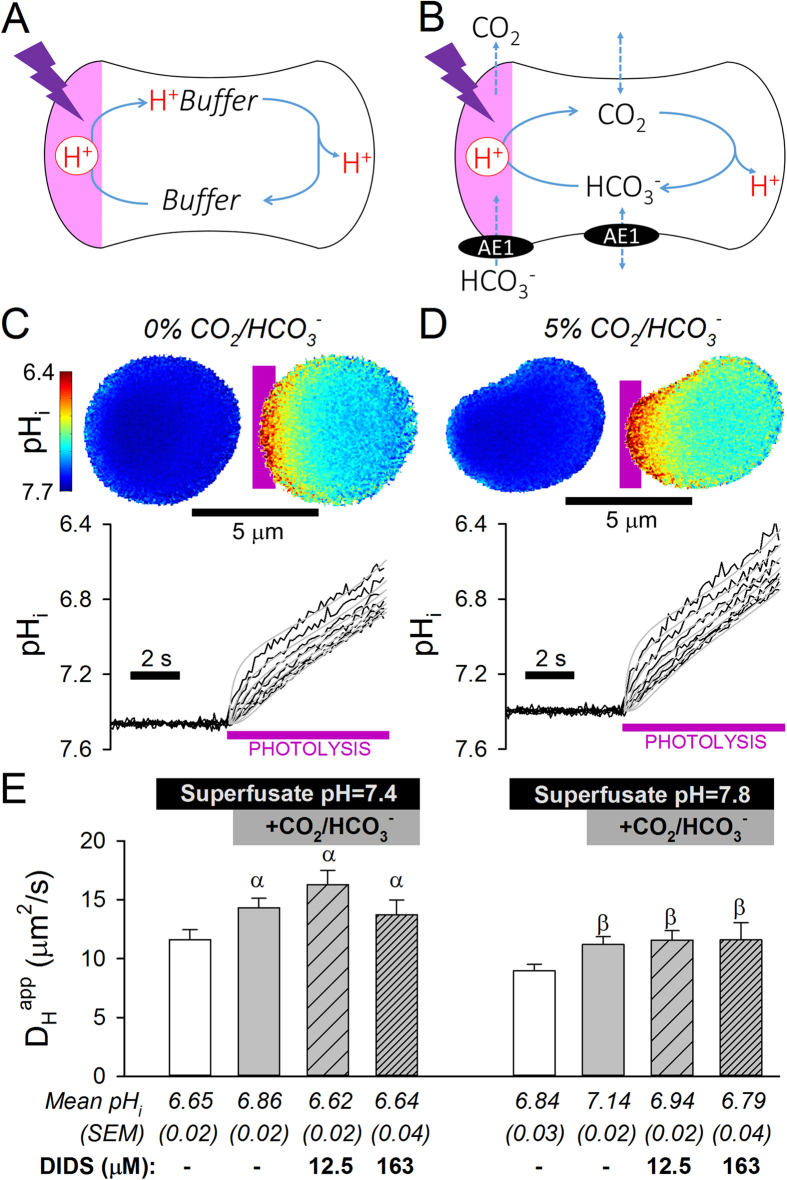
H^+^ mobility in the cytoplasm of human RBCs is a read-out of CO_2_/HCO_3_^−^ diffusivity. (**A)** Buffer (e.g. hemoglobin) facilitated H^+^ diffusion from a local H^+^-uncaging site (photolysis of 6-nitroveratraldehyde). **(B)** H^+^ diffusion facilitated additionally by CO_2_/HCO_3_^−^. Membrane HCO_3_^−^ permeability is set by AE1 (anion exchanger 1) activity. **(C)** Human RBC superfused in CO_2_/HCO_3_^−^-free buffer (at pH 7.8). H^+^ ions diffuse slowly from uncaging site, as shown by maps of intracellular pH (pH_i_) before and after 2 s of uncaging and pH_i_ time courses in regions of interest (ROIs) at increasing distance from the uncaging site. The width of each ROI was 1/10^th^ of RBC’s major diameter; data for ROIs 1 (uncaging site), 2, 3, 4, 5, 6 and 8 shown. Grey curves are best fit using a diffusion equation. **(D)** Experiment on RBCs superfused with 5% CO_2_/HCO_3_^−^ (at pH 7.8). The diffusion equation is solved using the finite element method which takes fully into account differences in cell outline, as observed between the cells shown in panels C and D. **(E)** Apparent H^+^ diffusion coefficients (D_H_^app^; mean ± SEM of 10–35 cells), showing the small effect of the CO_2_/HCO_3_^−^ buffer-shuttle. Where indicated, DIDS was added to block AE1. Symbols α and β denote significance (P < 0.05; P < 0.02) compared to measurements in the absence of CO_2_/HCO_3_^−^.

CO_2_ cannot be imaged dynamically inside a single intact RBC, but its acidic chemistry allows pH-sensitive fluorescent dyes, such as cSNARF1^20^, to monitor its diffusion. H^+^ ions are highly buffered in cells, therefore their apparent cytoplasmic diffusivity (D_H_^app^) is determined exclusively by the mobility of H^+^-carrying buffers (Fig. 1A,B)^21^,^22^. Based on this biophysical principle, it is possible to describe the diffusive properties of cytoplasmic buffers from measurements of H^+^ dynamics in intact cells^18^,^25^. To determine D_H_^app^, a local source of H^+^ ions was produced in cytoplasm by photolytic uncaging from the membrane-permeant H^+^-donor 6-nitroveratraldehyde (NVA)^23^ once every 0.13 s. This generates an acidic microdomain which dissipates at a rate determined by the buffers’ concentrations, reaction kinetics with H^+^ ions, and diffusion coefficients. To follow the progress of buffer-facilitated H^+^ diffusion, pH-sensitive cSNARF1 fluorescence was imaged confocally across the RBC’s horizontal plane at intervals between H^+^ uncaging (e.g. Fig. 1C,D)^20^. Provided that membrane transport of H^+^ and HCO_3_^−^ ions is slow or inactivated, best-fitting these spatio-temporal [H^+^] data with a diffusion equation (derived previously^18^,^23^,^26^) gives a robust measure of D_H_^app^.

During experiments, continuous superfusion of cells controls for temperature and CO_2_/HCO_3_^−^ concentration^20^. In RBCs superfused with CO_2_/HCO_3_^−^-free solution, H^+^ diffusion is facilitated solely by hemoglobin (Fig. 1A). Under these conditions, D_H_^app^ reports H^+^ diffusion facilitated by the translation and rotation of hemoglobin molecules^27^. When RBCs are superfused with CO_2_/HCO_3_^−^-containing solution, cytoplasmic diffusion of H^+^ ions is additionally facilitated by CO_2_/HCO_3_^−^, thus D_H_^app^ reports the combined effects of hemoglobin and CO_2_/HCO_3_^−^ (Fig. 1B). Since these two buffer-shuttles are additive, the effect on D_H_^app^ of introducing CO_2_/HCO_3_^−^ into cytoplasm is a read-out of the turnover of the CO_2_/HCO_3_^−^ buffer-shuttle. High carbonic anhydrase (CA) activity in RBCs ensures that the CO_2_/HCO_3_^−^ buffer-shuttle is *not* meaningfully rate-limited by chemical reactions; instead, CO_2_/HCO_3_^−^-facilitated H^+^ diffusion is strongly dependent on the cytoplasmic diffusion coefficients of CO_2_ gas and HCO_3_^−^ ions (D_CO2_, D_HCO3_). Thus, the first step in quantifying D_CO2_ and D_HCO3_ is to probe the CO_2_/HCO_3_^−^-dependent and independent components of D_H_^app^.

### CO_2_/HCO_3_^−^ weakly facilitates cytoplasmic H^+^ diffusion in the cytoplasm of human RBCs

CO_2_/HCO_3_^−^-independent D_H_^app^ was measured in human RBCs superfused at 37 °C with CO_2_/HCO_3_^−^-free normal Tyrode (0NT) solution containing 1 mM NVA. Photolysis acidifies the cytoplasm at ~0.1 pH units/s, therefore D_H_^app^ measurements correspond to a mildly acid-shifted cytoplasmic pH (pH_c_). To probe D_H_^app^ over a range that encompasses physiological pH_c_, experiments were performed in superfusates at pH 7.4 or 7.8. Membrane transport of HCO_3_^−^ by AE1 is largely inactivated in the absence of CO_2_/HCO_3_^−^, therefore uncaged H^+^ ions are retained in cytoplasm. Figure 1C shows the slow spread of H^+^ ions through the cytoplasm of a human RBC in the absence of CO_2_/HCO_3_^−^. Measured D_H_^app^ (Fig. 1E) was three orders of magnitude slower than in water (1.2 × 10^4^ μm^2^/s)^28^. Immature RBCs, or reticulocytes, have been proposed to contain small histidine derivatives, such as carnosine^29^, which could facilitate H^+^ diffusion alongside hemoglobin. However, D_H_^app^ in cells identified positively by Thiazole Orange staining as reticulocytes was no different to D_H_^app^ in Thiazole Orange-negative (mature) RBCs (Fig. S1). Thus, the concentration of small-molecule histidine derivatives in reticulocytes is not sufficient to meaningfully influence H^+^ diffusivity.

Next, experiments were performed on RBCs superfused with CO_2_/HCO_3_^−^-buffered NT (BNT) containing 5% CO_2_ and either 22 mM HCO_3_^−^ (for pH 7.4) or 55 mM HCO_3_^−^ (for pH 7.8; Fig. 1D; solution osmolality was corrected to 296 mOsm/kg by balancing [NaCl]). If D_CO2_ were rapid, the CO_2_/HCO_3_^−^ buffer-shuttle would accelerate D_H_^app^ substantially and collapse pH gradients; however, introducing CO_2_/HCO_3_^−^ into cytoplasm increased D_H_^app^ by only 2–3 μm^2^/s (Fig. 1E), indicating that CO_2_/HCO_3_^−^ has a limited capacity to facilitate H^+^ diffusion, comparable to that of hemoglobin. Activation of AE1 in CO_2_/HCO_3_^−^-containing superfusates may, in principle, compromise the accuracy of D_H_^app^ measurements. However, D_H_^app^ estimates were unaffected by blocking AE1 with 12.5 μM or 163 μM 4,4′-diisothiocyano-2,2′-stilbenedisulfonic acid (DIDS; Fig. 1E), indicating that the magnitude of membrane HCO_3_^−^ transport is not sufficient to short-circuit the cytoplasmic CO_2_/HCO_3_^−^ buffer-shuttle (NB: even the lower dose of DIDS was sufficient to inhibit AE1; Fig. S2). Cytoplasmic CA activity was not inactivated by DIDS, NVA or its photolytic derivative (Fig. S3), therefore low D_H_^app^ was not an erroneous result of inhibited CO_2_/HCO_3_^−^ reaction kinetics.

### Solute diffusion in RBC cytoplasm is restricted by the high concentration of hemoglobin

**Figure 2:**
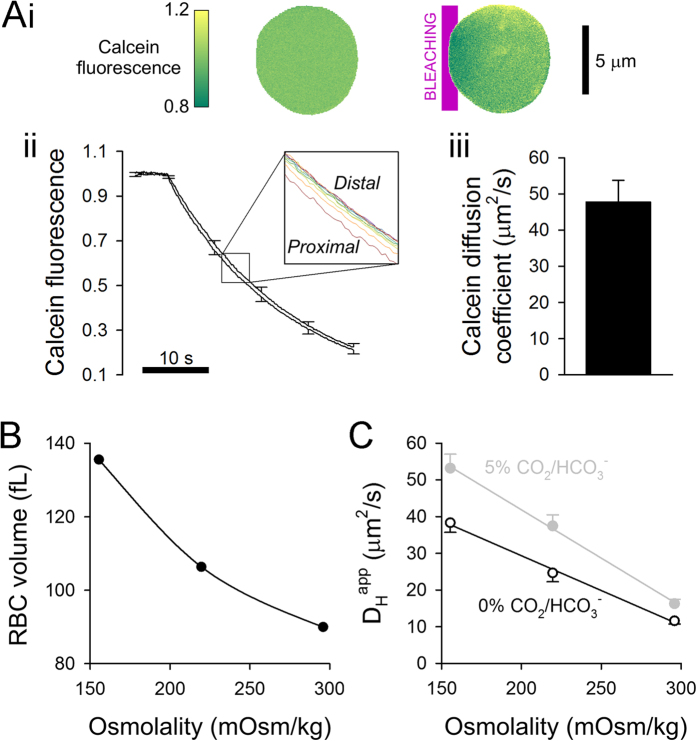
Hemoglobin concentration restricts cytoplasmic diffusivity. **(A)** Probing the diffusive tortuosity of RBC cytoplasm using calcein. (i) Localized bleaching (purple band) of calcein in intact human red blood cells (mean ± SEM of 16 cells) with a high-intensity 488 nm laser evoked a diffusive flux of calcein from non-bleached regions. Fluorescence maps normalized to the cell-averaged signal before bleaching *(left)* and after 20 s of localized bleaching *(right)*. (ii) Calcein diffusivity was derived by best-fitting fluorescence time courses averaged in 10 regions of interest (ROIs; defined in the same way as for H^+^ uncaging experiments). (iii) Calcein diffusion coefficient (mean ± SEM of 16 cells). **(B)** Osmotic swelling of human RBCs (flow cytometry repeated thrice; >50,000 cells each) dilutes hemoglobin concentration. **(C)** Apparent H^+^ diffusivity (D_H_^app^) increases as hemoglobin concentration is reduced, but the relationship is steeper in the presence of CO_2_/HCO_3_^−^ (plus 12.5 μM DIDS), indicative of increasing diffusive freedom for CO_2_/HCO_3_^−^ to facilitate H^+^ mobility (mean ± SEM of 9–28 cells).

To investigate if slow cytoplasmic diffusivity is unique to CO_2_/HCO_3_^−^, the mobility of calcein (a fluorescent marker) was measured in intact human RBCs. Applying a high-intensity laser beam every 0.13 s to one end of the cell bleaches calcein locally, and drives a diffusive redistribution of fluorescence signal, which was imaged throughout the cell at intervals between bleaching events. Time delays in calcein fluorescence measured at different distances from the bleaching region provide a readout of cytoplasmic diffusivity (D_calc_), measured to be 47.8 ± 5.95 μm^2^/s, i.e. 13-fold lower than in water (~600 μm^2^/s)^30^ (Fig. 2A). This result indicates that RBC cytoplasm is a highly tortuous environment for diffusion.

Hemoglobin, which occupies a quarter of human RBC volume^20^, is likely to impose a substantial tortuosity to the movement of solutes in cytoplasm. Loosening hemoglobin density is expected to accelerate small-molecule diffusion, and this was tested in osmotically-swollen cells (Fig. 2B). RBCs were first pre-equilibrated in, and then superfused with 155 or 220 mOsm/kg solutions (prepared by reducing [NaCl]). In the absence of CO_2_/HCO_3_^−^, cytoplasmic dilution increased D_H_^app^ (Fig. 2C), presumably because of less restricted translational and rotational movements of H^+^-carrying hemoglobin molecules^27^. The relationship between D_H_^app^ and osmolality was steeper after introducing a constant concentration of CO_2_/HCO_3_^−^ into cytoplasm (Fig. 2C). This finding indicates that at lower hemoglobin density, CO_2_/HCO_3_^−^ is more effective in facilitating H^+^ diffusion. This occurs despite CA activity dilution, adding further evidence that H^+^ diffusion is not reaction-limited (i.e. even after 2-fold dilution, CA activity remains very high).

**Figure 3:**
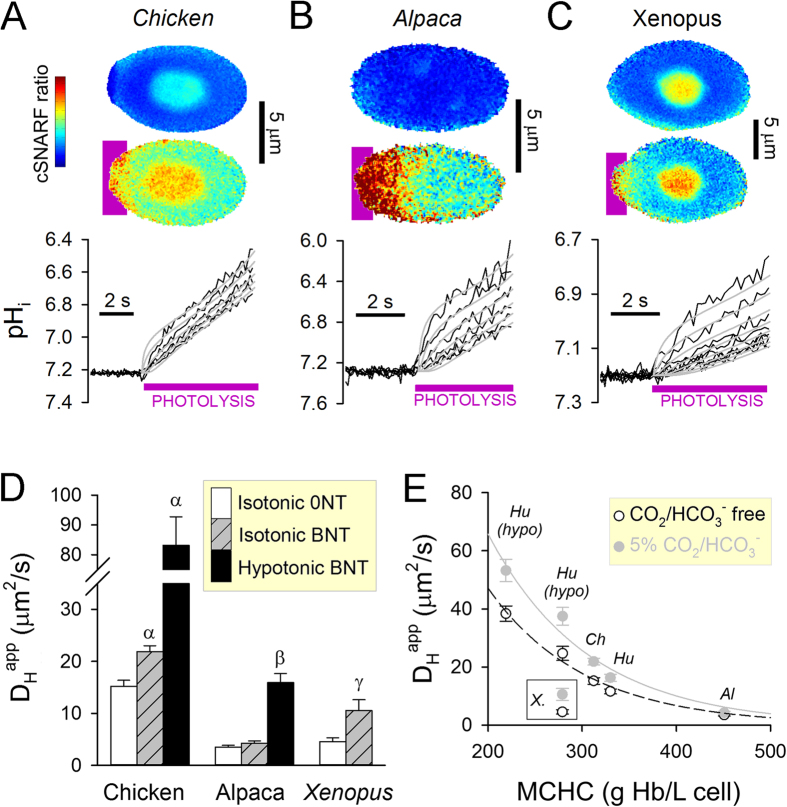
Measuring cytoplasmic H^+^ mobility in RBCs from different animal species. **(A**,**B)** H^+^ diffusion measurements in chicken and alpaca RBCs were performed according to the protocol for human RBCs (superfusate pH 7.8 and 37 °C). **(C)** For cold-blooded *Xenopus* RBCs, superfusates were at pH 7.5 and 25 °C. N.B.: For measuring fluorescence in ROIs, the signal from the nuclei was excluded. Measured pH_i_ time courses (*black;* in ROIs 1, 2, 3, 4, 5, 6, 8) are superimposed with best-fit time courses *(grey)*, determined by the least-squares method. **(D)** D_H_^app^ in chicken, alpaca and *Xenopus* RBCs measured in isotonic CO_2_/HCO_3_^−^-free buffer (0NT; mean ± SEM of 14, 21, 19 cells), isotonic CO_2_/HCO_3_^−^-containing buffer (BNT; mean ± SEM of 18, 25, 13 cells) or hypotonic BNT (mean ± SEM of 7, 19 cells). Superfusates were at pH 7.8 and 37 °C for mammals, and pH 7.5 and 25 °C for *Xenopus*. Osmotic swelling increases D_H_^app^ (not tested in *Xenopus* RBCs due to osmotic fragility). Symbols α, β and γ denote significance (P < 0.0005) compared to measurements in isotonic 0NT. **(E)** D_H_^app^ decreases with increasing mean corpuscular hemoglobin concentration (MCHC). *Hu*-human, *Ch*-chicken, *Al*-alpaca, *X.*-*Xenopus*; hypo: osmotically-swollen cells. The facilitatory effect of CO_2_/HCO_3_^−^ on D_H_^app^ diminishes at higher MCHC.

To explore the effect of naturally occurring differences in hemoglobin concentration on D_H_^app^, measurements were performed on RBCs from chicken, alpaca and *Xenopus* (Fig. 3A–C). Previous studies have detected carnosine in nucleated erythrocytes^31^, which may augment D_H_^app^ in chicken and *Xenopus* RBCs by acting as a mobile buffer alongside hemoglobin and CO_2_/HCO_3_^−^. However, using a modification of Pauly’s assay, levels of small-molecule histidine derivatives were in the sub-millimolar range, with the highest levels detected in chicken RBCs (0.5 mM; Fig. S4). Over this low concentration range, histidine-containing small molecules cannot meaningfully increase D_H_^app^, therefore hemoglobin and CO_2_/HCO_3_^−^ remain the principal H^+^-carriers. To measure D_H_^app^, alpaca and chicken RBCs were superfused in solution at 37 °C and pH 7.8, whereas *Xenopus* cells were superfused at room temperature and pH 7.5 to match their normal physiology. Nuclear regions in *Xenopus* and chicken RBCs were excluded in the analysis of pH (Hoechst 33342-positive nuclear areas also had higher intensity of cSNARF1 fluorescence, likely reflecting the ambient physicochemical environment of nucleoplasm; Fig. S5). Compared to human RBCs, chicken and *Xenopus* RBCs are larger and have a lower MCHC, whereas alpaca RBCs are smaller but with higher MCHC (Table S1). High CA activity was detected in hemolysates from all species studied, although it was lower in alpaca and *Xenopus* compared to humans (Fig. S6). Introducing CO_2_/HCO_3_^−^ into cytoplasm increased D_H_^app^ in RBCs from chicken and *Xenopus*, but not alpaca (Fig. 3D). Hypotonic swelling (i.e. loosening hemoglobin density) increased D_H_^app^ further in chicken and alpaca RBCs (NB: *Xenopus* RBC are too fragile for this experiment). Figure 3E summarizes the relationship between D_H_^app^ and MCHC; with the exception of cold-blooded *Xenopus*, all data-points followed an exponentially-declining relationship. In summary, the facilitatory effect of CO_2_/HCO_3_^−^ on D_H_^app^ was attenuated at higher MCHC, consistent with hemoglobin-imposed tortuosity.

### Calculated CO_2_ diffusivity in cytoplasm is slow and dependent on hemoglobin concentration

Cytoplasmic H^+^ diffusivity is related mathematically^21^,^22^ to the concentration, mobility and protonation/deprotonation kinetics of hemoglobin and CO_2_/HCO_3_^−^. In this system, all variables except for CO_2_/HCO_3_^−^ mobility (D_CO2_, D_HCO3_), are known, therefore an algorithm can be designed to calculate D_CO2_ and D_HCO3_ from D_H_^app^ measurements. Since CO_2_ diffuses 46% faster than HCO_3_^−^ (a difference attributable to molecular size)^2^, D_CO2_ and D_HCO3_ are numerically constrained to one another, reducing the system of equations to one unknown. This algorithm, described in more detail in the Supplement, performs simulations for a range of different test-values of D_CO2_ (and D_HCO3_) and for each generates a predicted D_H_^app^. Least-squares best-fitting this output to the experimentally-determined D_H_^app^ derives D_CO2_ (and D_HCO3_). The ‘known’ variables required to run these simulations were obtained as follows. Firstly, the concentration of hemoglobin was obtained from MCHC measurements^20^,^32^, and the cytoplasmic concentration of CO_2_/HCO_3_^−^ was calculated using the Henderson-Hasselbalch equation from extracellular [CO_2_] and [HCO_3_^−^] and the measured transmembrane pH gradient. Secondly, hemoglobin reaction kinetics were assumed to be instantaneous, whereas the reactions of CO_2_/HCO_3_^−^ were modelled kinetically using data for CA activity, hydration and dehydration rate constants. Finally, hemoglobin-facilitated H^+^ diffusivity is equal to D_H_^app^ measured in CO_2_/HCO_3_^−^-free media.

**Figure 4:**
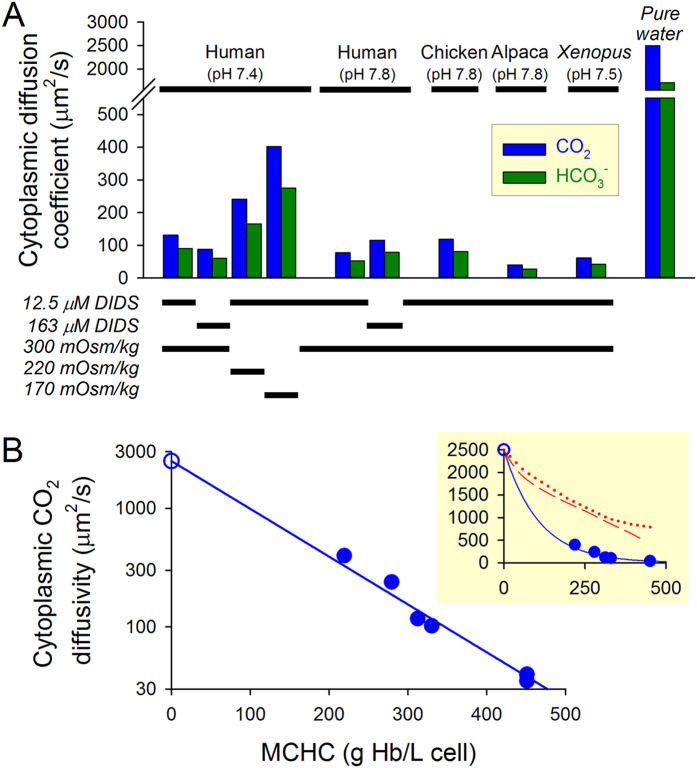
CO_2_ diffuses slowly in RBC cytoplasm. **(A)** Cytoplasmic CO_2_ and HCO_3_^−^ diffusion coefficients calculated from D_H_^app^ (37 °C for human, chicken and alpaca, 25 °C for *Xenopus*) compared to data for pure water. **(B)** Cytoplasmic CO_2_ diffusivity fitted to an exponentially-declining function of mean corpuscular hemoglobin concentration (MCHC). Filled circles: data from human, chicken and alpaca RBCs; open circle: data for water (all 37 °C). Cytoplasmic CO_2_ diffusivity halves for every 75 g/L increase in MCHC. *Inset:* plot on linear y-axis, with data from ref. 2 (dashed line) and ref. 14 (dotted line) for gas diffusion coefficients normalized to CO_2_ diffusivity in water (2500 μm^2^/s).

Cytoplasmic D_CO2_ and D_HCO3_ were found to be substantially lower than the rates measured in water (Fig. 4A) and were inversely correlated with MCHC (Figs 4B and S7). For example, CO_2_ diffusivity in human RBC cytoplasm was 5% of the rate in water. The extent to which hemoglobin restricts small-molecule diffusion is substantially greater than previous estimates (inset to Fig. 4B; e.g. for human RBCs, the difference is one order of magnitude). Thus, highly restricted diffusivity of CO_2_ gas and HCO_3_^−^ anions in RBC cytoplasm is a hitherto unrecognized rate-limiting step in the process of gas exchange.

## Discussion

Chemical reactions and membrane transport are recognized to be critically important in determining the rate of gas exchange, and their experimental characterization has informed our current model of RBC physiology. The results of this study indicate that diffusion inside RBCs is a strongly rate-limiting factor for gas turnover. We find that RBC cytoplasm is a highly tortuous environment that substantially restricts the movement of solutes, consistent with the uniquely high microviscosity inside RBCs^33^. In terms of resistances to overall gas transport^34^, the component attributable to RBC cytoplasm has hitherto been underestimated, and so it is plausible that the other resistances in series (imposed by membranes and extracellular unstirred layers) have been exaggerated in earlier analyses^3^.

The extent to which the cytoplasm of intact human RBCs restricts gas diffusion is greater than estimated previously from cell-free solutions^12^,^15^. This may relate to the significantly slower rotational diffusion of hemoglobin inside RBCs compared to solution^33^, which argues that encapsulation in a membrane produces a more rigid lattice of macromolecules. It is also possible that diffusivity measurements in cell-free solutions may have been over-estimated as a result of convective currents of the medium, which are much less likely to occur inside cells. Of note is that our measured effect of hemoglobin on CO_2_ diffusivity is quantitatively similar to the effect of an unrelated macromolecule, Ficoll70, on rhodamine green diffusion^35^,^36^. Thus, hemolysates and hemoglobin-solutions may have only a limited ability to fully recapitulate the biophysical properties of intact RBC cytoplasm.

The cytoplasm of human RBCs reduces CO_2_ diffusivity by a factor of 23 and calcein diffusivity by 13-fold. The difference in tortuosity imposed on CO_2_ and calcein may relate to the solute’s reactivity with hemoglobin. CO_2_ binds weakly and reversibly to hemoglobin^2^ (forming carbamino-hemoglobin), which will retard CO_2_ translational diffusion. In contrast, the highly negatively charged calcein will not interact with hemoglobin in this way. Also, because the CO_2_ molecule (radius 2 Å) is 50-fold smaller than the calcein molecule (radius 7.4 Å), CO_2_ may be able interact more intimately with the surface of the hemoglobin (radius 27.5 Å). Consequently, CO_2_ may experience a more tortuous path around hemoglobin, compared to calcein.

**Figure 5:**
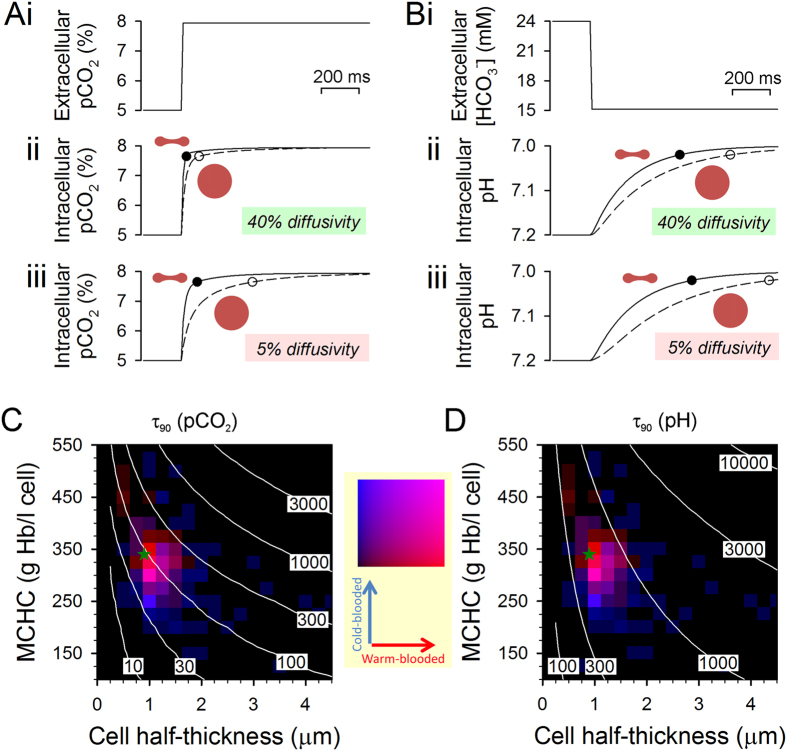
Results of mathematical simulations. **(A)** Time-to-complete 90% equilibration (τ_90_) of pCO_2_ inside RBC in response to an increase in ambient [CO_2_] (a respiratory acidosis of 7.2). (i) Extracellular pCO_2_. (ii) Simulations with fast D_CO2_ diffusivity: 40% of rate in water, as determined in cell-free solutions. Intracellular pCO_2_ in a flattened human RBC *(continuous line)* and a hypothetical spherical version of the same diameter *(dashed line)*. Circles indicate τ_90_. (iii) Simulations repeated with more restricted D_CO2_ diffusivity: at 5% of rate in water. The benefit of a flattened shape is more apparent with slow D_CO2_. **(B)** Time-to-complete 90% (τ_90_) of acid uptake by an RBC in response to a decrease in ambient [HCO_3_^−^] (a metabolic acidosis of 7.2). (i) Extracellular [HCO_3_^−^]. (iii) Simulations with fast D_CO2_ for flattened *(continuous line)* and spherical *(dashed line)* cells. Circles indicate τ_90_. (iii) Simulations repeated with more restricted D_CO2_. **(C)** Heat map showing binned data on MCHC and RBC half-thickness data from >250 species of cold- and warm-blooded animals (see inset for look-up table). Superimposed contours (units: ms) show model predictions for time-to-complete 90% equilibration τ_90_ of pCO_2_ in RBCs, based on human RBC data modified accordingly for thickness, MCHC and hence diffusivity of CO_2_, HCO_3_^−^ and hemoglobin. Most species fall in the range 0.03 s < τ_90_ < 0.3 s. **(D)** Heat map superimposed with model predictions for time-to-complete 90% (τ_90_) acid uptake into RBCs, based on human RBC data modified according for thickness, MCHC and hence diffusivity of CO_2_, HCO_3_^−^ and hemoglobin. Most species fall in the range 0.3 s < τ_90_ < 1 s.

Since diffusivity, path-length and cell shape are inter-related, our measurements are an important addition to our understanding of RBC form and function. In light of highly restricted diffusivity, the advantage of flattening an RBC can be explained in terms of minimizing critical time delays, which are proportional to the square of distance. To explore this proposal, a mathematical model (described more fully in the Supplement) simulated the time course of CO_2_ penetration into human RBCs entering a hypercapnic (8% CO_2_) environment (Fig. 5Ai). Predictions for normal human RBCs (half-thickness 0.9 μm; diameter 8 μm) were compared to those for a hypothetical spherical variant (diameter 8 μm). Using D_CO2_ data derived previously from cell-free solutions (40% of diffusivity in water, labelled here as ‘fast’), the time-to-reach 90% of the equilibrium state (τ_90_) for CO_2_ partial pressure (pCO_2_) was adequately rapid in both the flat (0.03 s) and spherical (0.09 s) geometry (Fig. 5Aii). Simulations using the presently determined value of D_CO2_ (5% of diffusivity in water, i.e. ‘slow’) showed a dramatically prolonged τ_90_ for pCO_2_ equilibration: 0.09 s for a flat RBC and 0.38 s for its spherical variant (Fig. 5Aiii). The later delay is incompatible with efficient gas exchange with faster blood flows (e.g. in exercise). Thus, the critical importance of RBC thickness for efficient gas exchange becomes apparent only when *slow* cytoplasmic diffusivity is considered. The cytoplasmic restrictions imposed on CO_2_ movement may also apply to O_2_ diffusion, therefore RBC thickness could also be important in determining the rate of O_2_ exchange.

Another consequence of profoundly restricted CO_2_ and HCO_3_^−^ diffusion is very slow H^+^ ion mobility in RBC cytoplasm. Remarkably, H^+^ diffusivity inside RBCs is the *slowest* among all ions studied in cells; this is somewhat surprising given that RBCs experience the highest acid-base turnover in the body^26^. H^+^ ions are a powerful signal that regulates O_2_ binding to hemoglobin through the Bohr^24^ and Root Effects^37^,^38^, but this response is only effective if the RBC’s mean cytoplasmic pH (pH_RBC_) is able to track changes in ambient pH (e.g. during the transit through acidic microvasculature of contracting muscles). Slow cytoplasmic H^+^ diffusion thus places an additional constraint on RBC thickness, which was analyzed, using the model, in terms of the pH_RBC_ response to an ambient metabolic acidosis (Fig. 5Bi). Assuming the higher D_CO2_ value, a flattened RBC will acidify faster than a spherical cell, but in both cases, τ_90_ was under 1 s (0.48 s for flat; 0.75 s for spherical; Fig. 5Bii). Using the lower D_CO2_ value measured herein, τ_90_ would increase to 0.54 s and 0.95 s for flat and spherical cells, respectively (Fig. 5Biii). The delay associated with spherical geometry would result in only a partial manifestation of the Bohr Effect, and consequently suboptimal distribution of O_2_ to tissues. Thus, in order for O_2_ release from RBCs to respond adequately to changes in ambient pH, the cell must acquire a flattened form.

Circulating human RBCs undergo a dramatic velocity-dependent deformation (e.g. parachute-like shape) as they transit through the microvasculature^39^,^40^,^41^. As a result of cytoplasmic re-distribution, the intracellular diffusion distances may modestly increase towards the leading edge of the cell and decrease at rear. Nonetheless, the parachute RBC retains an overall flattened form that is compatible with efficient gas exchange. This ability of flattened human RBCs to attain a parachute shape in capillaries may be critical for maintaining minimal cytoplasmic path-lengths for efficient gas exchange.

Our work also quantified the effect of MCHC on gas diffusion. There is considerable inter-species variation in MCHC (Table S2) which, at least in part, is driven by demand for O_2_-carrying capacity, but nonetheless appears to be capped at ~550 g/L. High MCHC is associated with raised microviscosity and resistance to blood flow^6^,^42^, which could underlie the biological limit of MCHC, although this can be compensated for by reducing RBC count. Here, we demonstrate that raising MCHC by 75 g/L halves D_CO2_ (Fig. 4B), an effect that is quantitatively similar to that of other macromolecules (e.g. rhodamine green diffusivity halves with every 88 g/L rise in Ficoll70 concentration)^36^. Thus, a physiological need to pack more hemoglobin into a RBC (e.g. observed in some altitude-adapted species, such as llamas and alpacas) comes at the cost of more restricted gas diffusivity. Cells adapted in this manner would need to be appropriately thinner in order to support efficient gas exchange. To investigate this hypothesis, we compiled data on RBC half-thickness (i.e. the shortest cytoplasmic path-length) and MCHC from >250 cold- and warm-blooded species (see Table S2; Fig. S8). Figure 5C shows a two-dimensional frequency histogram for RBC half-thickness and MCHC, superimposed with simulations for the time-to-reach 90% pCO_2_ equilibrium (τ_90_), determined using the model, shown in Fig. 5A, with appropriately varied MCHC (hence D_CO2_, D_HCO3_) and half-thickness. The majority of naturally-occurring MCHC/half-thickness combinations fell in the τ_90_ range of 0.03−0.3 s, i.e. compatible with near-complete equilibration during typical capillary transit times. This model was also used to simulate τ_90_ for pH_RBC_ equilibration in response to ambient acidosis. The majority of naturally-occurring MCHC/half-thickness combinations were constrained to meet the criterion of τ_90_ < 1 s (Fig. 5D). We postulate that thick RBCs with high MCHC (i.e. upper right quadrant in Fig. 5C,D) are not normally found in nature because these would be associated with unacceptably slow gas exchange (τ_90_ for pCO_2_ equilibration >0.3 s) and an incomplete manifestation of the Bohr Effect (τ_90_ for pH_RBC_ equilibration >1 s). Membrane tension and, in some species, the presence of a nucleus limit the extent to which RBCs could be made thinner; we propose that this imposes a cap on MCHC. According to Fig. 4B, MCHC greater than 500 g/L would restrict CO_2_ diffusion by a factor of >100, requiring RBCs to be thinner than 1 μm; this may explain why such high hemoglobin levels are rare, despite pertinent selection pressures for increasing O_2_-carrying capacity. This line of reasoning may also explain why mature RBCs in mammals have lost their nucleus in a bid to become thinner.

The regulatory means by which normal RBCs meet the favorable MCHC/half-thickness criterion is likely to involve interactions within the set of gene loci (e.g. seventy-five in humans)^43^ that collectively determine RBC shape and hemoglobin content. Failure to attain a favorable combination of cell thickness and MCHC may impact on gas exchange efficiency. The analyses shown in Fig. 5C,D highlight a potential disadvantage of attaining spherical symmetry in diseases such hereditary spherocytosis^44^, where mean cytoplasmic path-length increases due to shape and also MCHC tends to be higher due to cellular dehydration^45^,^46^. These circumstances predict a less complete gas exchange and only a partial manifestation of the Bohr effect at higher blood flows, which may contribute towards the reduced exercise tolerance commonly observed in patients with hereditary spherocytosis^46^.

Restricted diffusion in RBC cytoplasm may have important physiological implications besides gas exchange at capillaries. Slow diffusion of nitric oxide in RBC cytoplasm, in addition to the immobilizing effect of hemoglobin nitrosylation, may be a requirement for confining its signaling cascade to a sub-membranous domain^47^. A dense network of proteins may restrict gas diffusion in non-erythroid cell types or subcellular structures^48^. The magnitude of macromolecule-restricted diffusion highlights the potential for cytoplasm to regulate gas fluxes, a feat considered thus far to be in the remit of cell membranes only.

In conclusion, we propose that the efficiency of gas exchange depends critically on RBC thickness because the high density of hemoglobin restricts the movement of small solutes. This restriction is substantially greater in intact RBCs, compared to cell-free hemoglobin solutions or hemolysates, which explains why the cytoplasmic mobility of gases, HCO_3_^−^ and H^+^ ions has previously been overlooked as a rate-limiting step in the gas exchange cascade. In addition to increasing the cell’s surface area/volume ratio for greater trans-membrane solute traffic and providing mechanical advantages for blood flow, the evolutionarily-conserved flattening of RBCs is an essential adaptation to minimize delays owing to slow cytoplasmic diffusion.

## Methods

### Red blood cells

Alpaca (*Vicugna pacos*) and chicken (*Gallus gallus*) blood were collected by trained staff from Seralabs (U.K.) and delivered on ice within 24 hours of collection. *Xenopus laevis* blood was collected by ventricular puncture of animals that have been sacrificed humanely by pithing in accordance with Schedule I of Animal (Scientific Procedures) Act 1986 carried out in a licensed facility at Oxford University. Human blood was obtained from two volunteers who gave formal and informed consent, in accordance with Central University Research Ethics Committee (CUREC) guidelines (reference: R46540/RE001, approved procedure #24), and data were fully anonymized and not traceable to the donor. All methods involving the use of blood were performed in accordance with ethical guidelines set by Oxford University, with appropriate risk assessments in place. All experimental protocols performed on collected human red blood cells are performed in accordance with Oxford University’s Central University Research Ethics Committee (CUREC) guidelines (reference: R46540/RE001, approved procedure #24). Additionally, all experimental protocols perform on animal and human red blood cells were performed in accordance with Oxford University’s Health and Safety regulations (OHS Policy Documents 1/03, 1/01, OHS 2/03, UGN S1/95). Clinical tests confirmed that mean corpuscular volume (MCV) and mean corpuscular hemoglobin concentration (MCHC) of donor blood were in the normal range (MCV: 88.2–90.9 fL, MCHC: 325–334 g/L). Blood samples were collected in tubes treated with heparin (*Xenopus*) or EDTA (all other species), spun-down at 4 °C (10,000 for 5 min for mammalian blood; 5,000 for 5 min for chicken blood; 1,000 for 10 min for *Xenopus* blood) to remove the supernatant and buffy coat. A sample of the fraction containing packed red blood cells was re-suspended in Hepes-buffered NT and used for confocal imaging or flow cytometry; the remainder was freeze-thawed twice to break up cell membranes, and used for measurements of hemoglobin concentration (HemoCue Hb 201plus), histidyl-containing small molecules (Pauly’s assay) and carbonic anhydrase activity.

### Confocal imaging

To image intracellular pH (pH_i_), cells were loaded with the acetoxymethyl (AM) ester of the pH-reported dye cSNARF1 for 60 minutes (*Xenopus*) or 10 minutes (all other species), and allowed to settle on a poly-L-lysine pretreated coverslip at the base of a superfusion chamber mounted on an inverted Zeiss Axiovert microscope that was coupled to a Zeiss LSM700 confocal system. Superfusates were delivered at 4 mL/min at 25 °C (*Xenopus*) or 37 °C (all other species). Cells were imaged with a x100 objective and stored as stacks of 256 × 256 pixel 8-bit bitmaps (53 nm pixel length). cSNARF1 was excited by a 555 nm laser line and fluorescence was collected at 580 ± 10 and 640 ± 10 nm. The fluorescence ratio was converted to pH using a calibration curve, obtained by a published method^20^. To image calcein, cells were loaded with calcein-AM for 10 minutes, and fluorescence (>515 nm) was excited with low intensity 488 nm laser line. Offline analysis was performed using ImageJ.

### H^+^ uncaging and calcein bleaching

Photolytic uncaging of H^+^ ions from 6-nitroveratralhyde^23^ (NVA; added to solutions at 1 mM) was evoked by scanning a region of interest (ROI) at one end of the RBC (ROI width equal to 1/10^th^ of RBC diameter) with 405 nm laser light, as shown in Fig. 1C,D. By alternating between regional H^+^ uncaging and whole-field pH_i_-imaging, the spatial dissipation of H^+^ ions from the uncaging site can be followed. Fluorescence maps were analyzed using ImageJ. Two stacks of images (580 nm and 640 nm fluorescence) were background-offset and fluorescence signal was averaged in ten ROIs of width equal to 1/10^th^ of the RBC diameter along x-axis and height equal to the RBC diameter in the y-axis. For *Xenopus* and chicken, the fluorescence from the nucleus was excluded from analysis. ROI1 represented the uncaging region. The ratio of ROI fluorescence at 580 and 640 nm was converted to [H^+^] time courses, which were fitted with a diffusion equation to obtain the apparent diffusion coefficient (D_H_^app^), according to an algorithm described previously^26^ and in the Supplement. Bleaching of calcein fluorescence was performed by a similar protocol to H^+^ uncaging. The excitation protocol alternated between high intensity laser for localized calcein bleaching and low intensity 488 nm laser for imaging cytoplasmic calcein. Time courses of calcein fluorescence, measured in ten ROIs, were fitted with a diffusion equation, similar to that described in the Supplement for analyzing D_H_^app^.

## Additional Information

**How to cite this article**: Richardson, S. L. and Swietach, P. Red blood cell thickness is evolutionarily constrained by slow, hemoglobin-restricted diffusion in cytoplasm. *Sci. Rep.* **6**, 36018; doi: 10.1038/srep36018 (2016).

## Supplementary Material

Supplementary Information
