# Supplementary material for: Red blood cell thickness is evolutionarily constrained by slow, hemoglobin-restricted diffusion in cytoplasm
Source: Sci Rep. 2016 Oct 25;6:36018. doi: 10.1038/srep36018 (PMC5078773; doi:10.1038/srep36018)
Supplement: Supplementary Information [file srep36018-s1.pdf]

Red blood cell thickness is evolutionarily constrained by slow, hemoglobin-restricted diffusion in cytoplasm

Sarah L. Richardson & Pawel Swietach

SUPPLEMENTARY MATERIALS AND METHODS

Solutions. (a) Internal buffer (for measuring carbonic anhydrase activity): 40 mM KCl, 100 mM K-gluconate, 1 mM $MgCl_2$, 20 mM Hepes, pH 8 at 4°C. (b) Normal Tyrode (NT) solution for warm-blooded animals (for superfusion and flow cytometry): CO_2/HCO_3^--free NT (0NT) contained 130 mM NaCl, 4.5 mM KCl, 1 mM $CaCl_2$, 1 mM $MgCl_2$, 20 mM Hepes, 11 mM glucose at pH 7.4 or 7.8 at 37°C. CO_2/HCO_3^--buffered NT (BNT) contained 125 mM NaCl (for pH 7.4) or 91.5 mM NaCl (for pH 7.8), 4.5 mM KCl, 1 mM $CaCl_2$, 1 mM $MgCl_2$, 11 mM glucose, 22 mM $NaHCO_3$ (for pH 7.4) or 55.5 mM $NaHCO_3$; BNT solutions were bubbled with 5% CO_2 at 37°C. Hypotonic solutions were made by reducing [NaCl] and osmolality was measured by a freezing point osmometer. (c) Normal Tyrode for *Xenopus* was modified to amphibian Tyrode[1]: NaCl and KCl were reduced by 45 mM and 2 mM respectively, $CaCl_2$ was raised by 1 mM in 0NT and BNT; $NaHCO_3$ in BNT was 26 mM, and pH was 7.5 at 25°C.

Pauly's assay. Previous studies have reported the presence of carnosine in reticulocytes [2] and nucleated erythrocytes [3]. To measure the levels of small, histidyl-containing molecules (such as carnosine), 5-fold diluted hemolysates were mixed 1:1 (v/v) with chloroform and centrifuged at 4°C at 13,400 rpm for 10 min to remove hemoglobin. The supernatant was collected and spun in 10 kDa filter tubes (4°C at 13,400 rpm for 10 min). The clear filtrate was reacted with Pauly's reagents in 96-well plates. Equal volumes of sodium nitrite (5% w/v) and sulfanilic acid (0.45 g in 4.5 ml 5 N HCl and 45 ml water) were incubated with the filtrate at 25°C for 5 min. Na_2CO_3 (10% w/v) was then added 2:1 (v/v). 5 min after the start of the reaction, absorbance of the product at 490 was probed by plate reader. Calibration was performed using solutions containing 50 g/L hemoglobin and between 0 and 10 mM carnosine.

Carbonic anhydrase activity. Hemolysates were diluted in internal buffer and aliquots of 0.67 mL were added to a stirred reaction chamber at 4°C. A Biotrode pH electrode measured solution pH at 1 Hz. Addition of 0.33 mL 100% CO_2-saturated water to the chamber triggered the

carbonic anhydrase (CA)-catalyzed reaction, which was quantified in terms of a hydration rate constant by best fit to a kinetic model [4], and expressed as a ratio to the spontaneous rate measured in the presence of the CA inhibitor acetazolamide (200 μM). Measurements were repeated over a series of dilutions, and activity was extrapolated to 100% hemolysate in order to estimate CA activity in the undiluted cytoplasmic environment at 4°C. Hemolysate dilution was determined as the ratio of literature values for MCHC to hemoglobin concentration in hemolysates (HemoCue Hb 201$^+$ hemoglobinometer).

Identifying reticulocytes. Human RBCs were loaded with Thiazole Orange (1:1000 dilution) for 30 min in PBS at 37°C and allowed to settle in the superfusion chamber. Fluorescence, excited at 488 nm, was measured at 515 nm. Reticulocytes were identified by fluorescence in discrete regions of the cell corresponding to nucleic acids (Fig. SAi). By co-loading cells with cSNARF1, reticulocyte pH_i could be measured because 555 nm-excited cSNARF1 fluorescence does not overlap with 488 nm-excited Thiazole Orange fluorescence.

Flow cytometry. Cells were loaded with calcein to allow positive identification of intact RBCs by gating for >515 nm fluorescence. Cell volume was inferred from the electronic volume reported with a Beckman Coulter Quanta SC flow cytometer, and calibrated against volume-standards [5].

Calculating the apparent cytoplasmic H^+ diffusion coefficient. The diffusion equation was solved using the finite element method in two dimensions over the cell's geometry, defined as the outline of fluorescence at a threshold equal to half the mean fluorescence signal at the center of the cell (nucleus, if present, was excluded in the mean). The location of uncaging was fixed to ROI1, and the rate of acid-loading was determined from the measured rate of $[H^+]$-rise best fitted to an exponential function. Reflection boundary conditions were imposed. The initial condition was uniform $[H^+]$. Simulations were run over a range of diffusion coefficients (D_H^{app}). The best-fit D_H^{app} was determined by least-squares fitting of simulation to the experimentally-determined time courses. For reference, H^+ diffusivity in water is 1.2×10^{-4} $\mu m^2/s$ at 37°C [6].

Calculating the cytoplasmic calcein diffusion coefficient. RBC geometry was defined as the outline of calcein fluorescence at a threshold equal to half the mean fluorescence signal in the center of the cell. The diffusion equation was solved with reflection boundary conditions. The initial condition was uniform concentration. A reaction term was introduced to gradually reduce fluorescence in the bleached area (ROI1); the rate of bleaching was determined by best-fitting the data to an exponential function. Simulations were run over a range of diffusion coefficients. Best-fit

calcein diffusivity was determined by least-squares fitting of simulation to experimentally-obtained time courses. For reference, calcein diffusivity in water is 604 $\mu m^2/s$ at 37°C [7-9].

Calculating cytoplasmic CO_2 diffusivity from D_H^{app} measurements. Since the CO_2/HCO_3^- buffer-shuttle facilitates H^+ diffusion, there is a mathematical relationship between the apparent H^+ diffusion coefficient (D_H^{app}) measured in the presence of CO_2/HCO_3^-, hemoglobin-facilitated H^+ diffusivity (D_{Hb}), intracellular CA activity (CA_i), and the diffusion coefficients of CO_2 and HCO_3^- (D_{CO2}, D_{HCO3}). D_{CO2} was constrained to D_{HCO3} in a 1.46:1 ratio [10]. A model of buffer-facilitated H^+ diffusion was developed to produce a unique relationship between D_{CO2} and D_H^{app} for each experimental condition and each species studied. Using this relationship, D_{CO2} (Fig. 3) was calculated from D_H^{app} measurements.

MCHC and MCV were referenced from Table S2. For osmotically-swollen cells, MCHC and MCV were corrected according to flow cytometry analyses. Mean cell thickness (h) was calculated as the ratio of MCV to cross-sectional area measured from the cell's fluorescence outline. D_{Hb} is equal to D_H^{app} measured in the absence of CO_2/HCO_3^- (Fig. 1). k_f and k_r are the uncatalyzed forward and reverse rate constants of CO_2 hydration at 37°C: 0.18 s^{-1} and 0.23 μM^{-1} s^{-1} [10,11]. CA activities (CA_i) were obtained from Fig. S6. The system of partial differential equations was solved for intracellular $[H^+]$ (u_1), $[HCO_3^-]$ (u_2) and $[CO_2]$ (u_3) over the long-axis of the cell perpendicular to the uncaging ROI between x=0 (near site of uncaging) and $x=L$ (where L is the length of the RBCs).

$$\frac{\partial u_1}{\partial t} = D_{Hb} \times \frac{\partial^2 u_1}{\partial x^2} + \frac{CA_i \times (k_f \times u_3 - k_r \times u_1 \times u_2) + J(x)}{\beta_{Hb}/(2.303 \times u_1)}$$

$$\frac{\partial u_2}{\partial t} = D_{HCO3} \times \frac{\partial^2 u_2}{\partial x^2} + CA_i \times (k_f \times u_3 - k_r \times u_1 \times u_2)$$

$$\frac{\partial u_3}{\partial t} = D_{CO2} \times \frac{\partial^2 u_3}{\partial x^2} - CA_i \times (k_f \times u_3 - k_r \times u_1 \times u_2) + \rho \times P_{CO2}^{app} \times (u_3^0 - u_3)$$

The rate of H^+ uncaging, J, was equal to the experimentally-determined acidification rate (Fig 1) and restricted to the area of uncaging (ROI1). Buffering due to hemoglobin (β_{Hb}) was calculated as 0.178×MCHC based on the finding that buffering capacity in human RBCs is 60.5 mmol/(L cell) at 340 g Hb/(L cell) [12,13]. ρ is the surface area-to-volume ratio equal to the inverse of cell half-thickness (h/2). P_{CO2}^{app} is the apparent permeability to CO_2 across the half-thickness of the cell (h/2), equal to $D_{CO2}/(h/4)$ i.e. diffusion across the mean path-length over the domain x=0 to $x=h/2$. Extracellular CO_2 (u_3^0) was set to 1.2 mM.

The simulated time-courses were then best-fitted to a simple diffusion equation (the same as that described above for fitting experimental $[H^+]$ time courses) to obtain D_H^{app}. This approach generated a D_{CO2}-D_H^{app} relationship for each species and experimental condition. CA activity in

human and chicken RBCs is very high (>10^4-fold acceleration of k_f; Fig. S6) and sensitivity analysis demonstrates that that the value of CA_i used in the model does not meaningfully affect the D_{CO2}-D_H^{app} relationship. CA_i was lower in alpaca RBCs compared to human cells (Fig. S6); however, even at this rate, the D_{CO2}-D_H^{app} relationship was not meaningfully sensitive to CA_i (e.g. raising CA_i in the model by a factor of 10 did not affect the D_{CO2} prediction for alpaca RBCs).

Simulating gas exchange in RBCs. Multiple steps of the gas exchange cascade introduce significant time delays. The sum of these delays must be less than 1 s in order to complete gas exchange during the RBC's brief capillary transit. Of the two blood gases, CO_2 takes longer to transfer in and out of the RBCs because the vast majority of this gas is transported as its chemically converted form, HCO_3^-. Human RBCs accelerate CO_2 fluxes with *(i)* exceptionally high CA activity (20,000-fold acceleration [14,15]) to catalyze CO_2–HCO_3^- chemical conversion, *(ii)* high AE1 activity to facilitate membrane transport of HCO_3^- (membrane permeability P_{HCO3}=18 μm/s [14,15]) and *(iii)* express putative gas channels to facilitate membrane permeation of CO_2 (membrane permeability P_{CO2}=1200 μm/s [14,15]). The explore the degree to which cytoplasmic gas diffusion delays gas exchange, a mathematical model of CO_2 loading into RBCs was developed. MCHC and cell half-thickness (*h*/2) were varied independently. Cytoplasmic diffusion coefficients of hemoglobin (D_{Hb}), CO_2 (D_{CO2}) and HCO_3^- (D_{HCO3}) were expressed as a function of MCHC according to experimental findings (see Fig. S7; D_{CO2} was constrained to D_{HCO3} in a 1.46:1 ratio [10]):

$$D_{Hb} = 415 \times \exp(-MCHC/93.7)$$

$$D_{CO2} = 2500 \times \exp(-MCHC/107.7)$$

$$D_{HCO3} = 1712 \times \exp(-MCHC/107.7)$$

Cytoplasmic CA activity (CA_i), P_{HCO3} and P_{CO2} were set to values for human RBCs, as referenced above. k_f and k_r are the uncatalyzed forward and reverse rate constants of CO_2 hydration at 37°C: 0.18 s^{-1} and 0.23 μM^{-1} s^{-1} [10,11]. The system of partial differential equations was solved for intracellular [H^+] (u_1), [HCO_3^-] (u_2) and [CO_2] (u_3) over the cell's half-thickness between x=0 (midline of cell) and x=h/2 (cell surface):

$$\frac{\partial u_1}{\partial t} = D_{Hb} \times \frac{\partial^2 u_1}{\partial x^2} + \frac{CA_i \times (k_f \times u_3 - k_r \times u_1 \times u_2) + J(x)}{\beta_{Hb}/(2.303 \times u_1)}$$

$$\frac{\partial u_2}{\partial t} = D_{HCO3} \times \frac{\partial^2 u_2}{\partial x^2} + CA_i \times (k_f \times u_3 - k_r \times u_1 \times u_2)$$

$$\frac{\partial u_3}{\partial t} = D_{CO2} \times \frac{\partial^2 u_3}{\partial x^2} - CA_i \times (k_f \times u_3 - k_r \times u_1 \times u_2)$$

The boundary conditions for u_1, u_2 and u_3 at x=0 were reflection and at x=h/2 were:

$$\frac{\partial u_1}{\partial x} = 0$$

$$\frac{\partial u_2}{\partial x} = \rho \times P_{HCO3} \times (u_2^0 - 10^{-0.2} \times u_2)$$

$$\frac{\partial u_3}{\partial x} = \rho \times P_{CO2} \times (u_3^0 - u_3)$$

The factor $10^{-0.2}$ in the boundary condition for HCO_3^- was included to simulate the 0.2 pH-unit difference between intra- and extracellular pH (intracellular more acidic) [5]; u_2^0 and u_3^0 were extracellular $[HCO_3^-]$=24 mM and $[CO_2]$=1.2 mM. Fig S9A/C/E show the results of simulations when u_3^0 is raised from 5% (1.2 mM) to 8% to simulate the effect of respiratory acidosis. Fig S9B/D/F show the results of simulations when u_2^0 is reduced from 24 mM to 15 mM to simulate the effect of metabolic acidosis.

Additional references

1. Stinner JN, Hartzler LK. Effect of temperature on pH and electrolyte concentration in air-breathing ectotherms. *J Exp Biol*. 2000;203(Pt 13):2065-2074.
2. Seely JE, Marshall FD. Carnosine levels in blood. *Experientia*. 1981;37(12):1256-1257.
3. Van Balgooy JN, Marshall FD, Roberts E. Carnosine in nucleated erythrocytes. *Nature*. 1974;247(5438):226-227.
4. Swietach P, Wigfield S, Cobden P, Supuran CT, Harris AL, Vaughan-Jones RD. Tumor-associated carbonic anhydrase 9 spatially coordinates intracellular pH in three-dimensional multicellular growths. *J Biol Chem*. 2008;283(29):20473-20483. 10.1074/jbc.M801330200.
5. Swietach P, Tiffert T, Mauritz JM, et al. Hydrogen ion dynamics in human red blood cells. *J Physiol*. 2010;588(Pt 24):4995-5014. 10.1113/jphysiol.2010.197392.
6. Swietach P, Zaniboni M, Stewart AK, Rossini A, Spitzer KW, Vaughan-Jones RD. Modelling intracellular H(+) ion diffusion. *Prog Biophys Mol Biol*. 2003;83(2):69-100.
7. Papadopoulos MC, Kim JK, Verkman AS. Extracellular space diffusion in central nervous system: anisotropic diffusion measured by elliptical surface photobleaching. *Biophys J*. 2005;89(5):3660-3668. 10.1529/biophysj.105.068114.
8. Yoshida N, Tamura M, Kinjo M. Fluorescence Correlation Spectroscopy: A New Tool for Probing the Microenvironment of the Internal Space of Organelles. *Single Molecules*. 2000;1:279.
9. Becker S, Zorec B, Miklavcic D, Pavselj N. Transdermal transport pathway creation: Electroporation pulse order. *Math Biosci*. 2014;257:60-68. 10.1016/j.mbs.2014.07.001.

10. Geers C, Gros G. Carbon dioxide transport and carbonic anhydrase in blood and muscle. *Physiol Rev*. 2000;80(2):681-715.

11. Hulikova A, Aveyard N, Harris AL, Vaughan-Jones RD, Swietach P. Intracellular carbonic anhydrase activity sensitizes cancer cell pH signaling to dynamic changes in CO2 partial pressure. *J Biol Chem*. 2014;289(37):25418-25430. 10.1074/jbc.M114.547844.

12. Cass A, Dalmark M. Equilibrium dialysis of ions in nystatin-treated red cells. *Nat New Biol*. 1973;244(132):47-49.

13. Dalmark M. Chloride and water distribution in human red cells. *J Physiol*. 1975;250(1):65-84.

14. Endeward V, Gros G. Extra- and intracellular unstirred layer effects in measurements of CO2 diffusion across membranes--a novel approach applied to the mass spectrometric 18O technique for red blood cells. *J Physiol*. 2009;587(Pt 6):1153-1167. 10.1113/jphysiol.2008.165027.

15. Endeward V, Musa-Aziz R, Cooper GJ, et al. Evidence that aquaporin 1 is a major pathway for CO2 transport across the human erythrocyte membrane. *FASEB J*. 2006;20(12):1974-1981. 10.1096/fj.04-3300com.

Red blood cell thickness is evolutionarily constrained by slow, hemoglobin-restricted diffusion in cytoplasm

Sarah L. Richardson & Pawel Swietach

SUPPLEMENTARY FIGURES

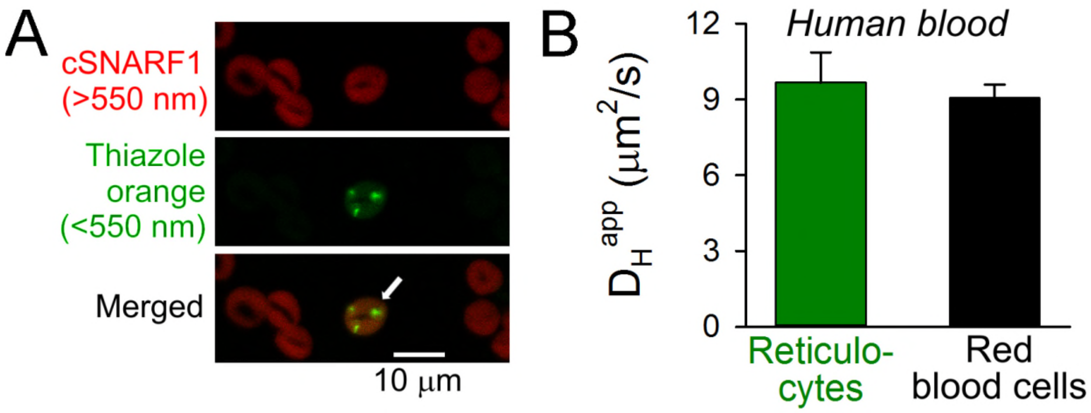

Fig. S1. *Testing for small histidine-containing molecules in reticulocytes.* **(A)** Fluorescence at >550 nm and <550 nm (488 nm laser excitation) in human RBCs loaded with cSNARF1 and Thiazole Orange. White arrow points to reticulocyte. **(B)** Measurements of H^+ diffusivity, performed according to the protocol shown in Fig 1A in 0NT (pH 7.8). Results show no significant difference between reticulocytes and Thiazole Orange-negative erythrocytes (mean±SEM of 15, 20 cells).

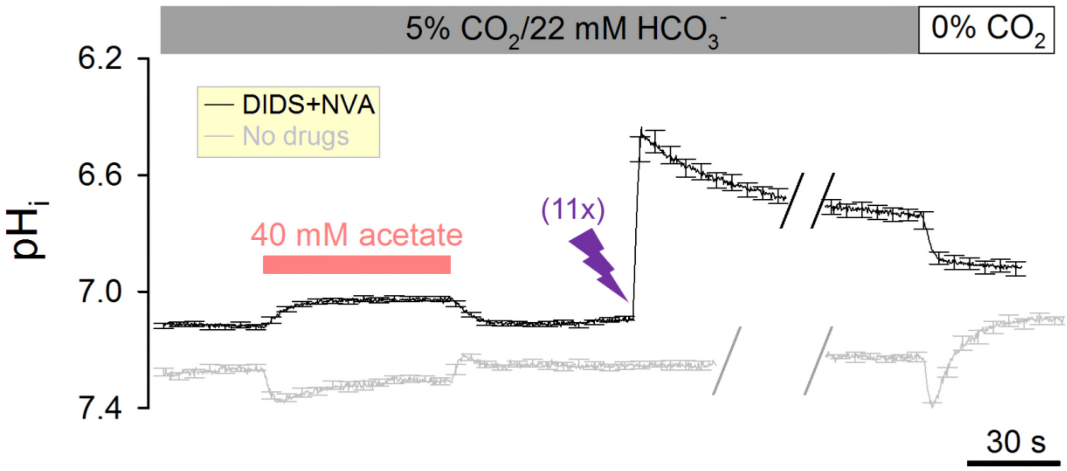

Fig. S2. *Testing the inhibitory effect of DIDS on anion exchanger 1 (AE1).* Intracellular pH (pH_i) response of human red blood cell to (1) 40 mM acetate pulse, (2) photolysis and (3) CO_2 wash-out. Experiments were performed in the presence (*black trace;* mean±SEM of 20 cells) or absence (*grey trace;* mean±SEM of 20 cells) of 12.5 μM DIDS and 1 mM NVA. (1) In the absence of drugs, the acetate pulse produces a transient alkalization (HCO_3^- uptake on AE1 evoked by the decrease in extracellular Cl^- in acetate-substituted solution), followed by slower acidification due to acetic acid entry across the lipid bilayer. The AE1-dependent component was blocked completely by DIDS. (2) In the presence of the caged H^+-compound NVA, exposure to a series of 11 pulses of UV light *(flash symbol)* deposited acid inside cells. The acid-load (0.65 pH-units) was retained in cytoplasm for >5 minutes, indicating that HCO_3^- uptake on AE1 (which would normally neutralize acidity) was inhibited by DIDS. (3) Removal of CO_2/HCO_3^- (replacement with Hepes) drives rapid CO_2 efflux from cells, reported as a rapid alkalization. This pH_i-response was not blocked by DIDS, NVA and its photolysis product, indicating that cell membranes remained permeable to CO_2 even in the presence of drugs (NB: the secondary acidification in drug-free conditions is due to HCO_3^- efflux on AE1).

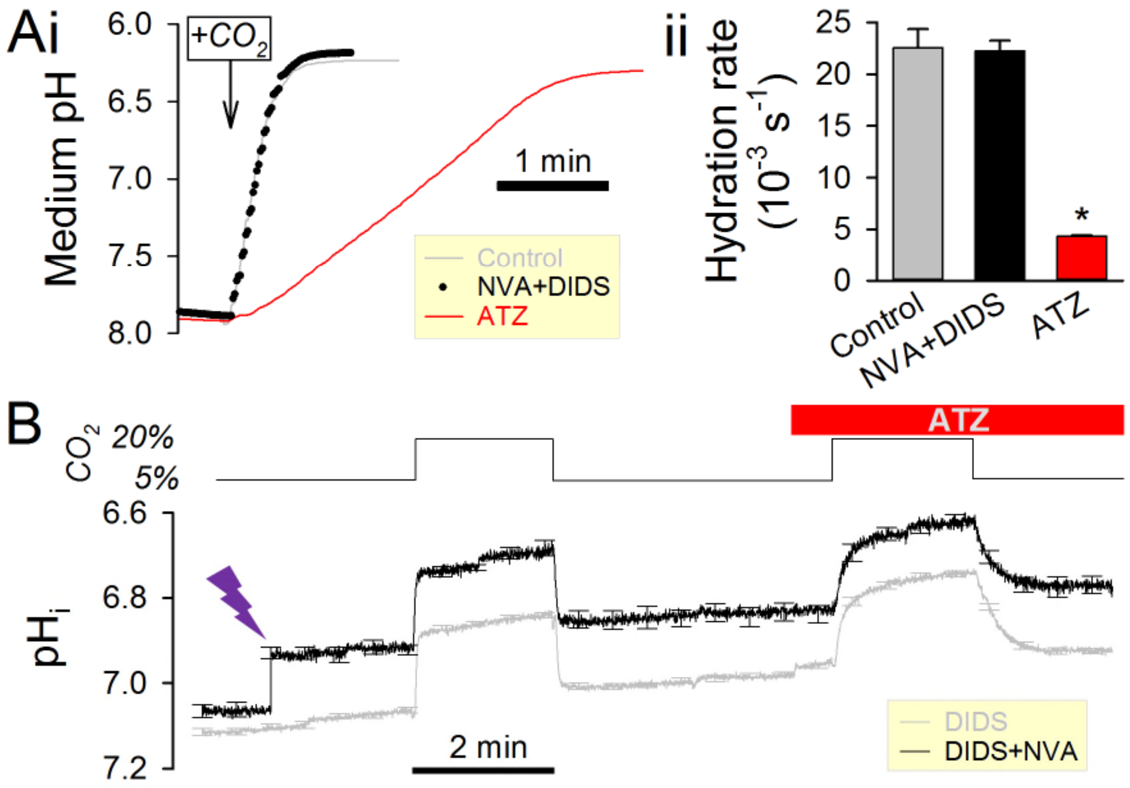

Fig. S3. *Measuring carbonic anhydrase activity.* **(A)** (i) Catalytic activity of native carbonic anhydrase (CA) isoforms in hemolysates diluted 1:1000 in internal solution, measured from the rate of CO_2 hydration at 4°C using medium pH as a read-out. The spontaneous rate was determined in the presence of 200 µM acetazolamide (ATZ), a CA inhibitor. (ii) Calculated hydration rate constants (1) (mean±SEM of 5 repeats each). * denotes a significant inhibition with ATZ ($P<10^{-4}$). 12.5 µM DIDS and 1 mM NVA did not affect CA activity in hemolysates. **(B)** Intact human RBCs superfused with BNT containing 12.5 µM DIDS, with (mean±SEM of 8 cells) or without (mean±SEM of 28 cells) 1 mM NVA. Whole-field H^+-uncaging *(flash symbol)* produced uniform cytoplasmic acidification in the presence of NVA only. Raising superfusate CO_2 partial pressure from 5% to 20% and back evoked in intracellular pH change, the rate of which gauges intracellular CA activity. The spontaneous rate was then determined in the presence of 500 µM ATZ. NVA, DIDS and the organic product of NVA photolysis did not inhibit intracellular CA activity in intact RBCs.

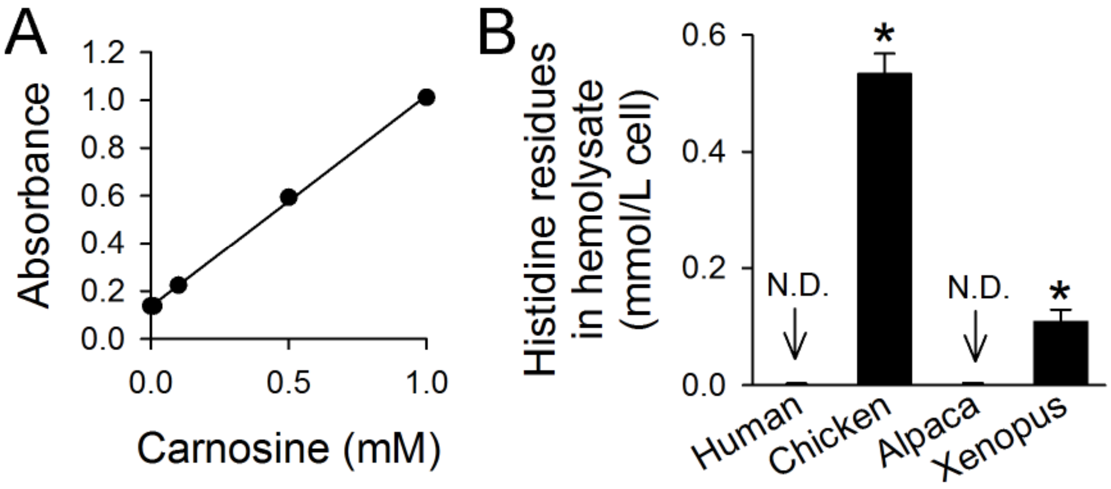

Fig. S4. *Testing for small histidine-containing molecules in reticulocytes.* **(A)** Calibration of Pauly's assay performed on purified carnosine. **(B)** Assay for small histidine-containing molecules in filtered hemolysates from human, chicken, alpaca and *Xenopus* packed RBCs. Data were expressed as mmol/(L cell) by normalizing to literature values for the mean corpuscular hemoglobin concentration (Table S1). N.D.-not detected. * denotes significant ($P<0.00002$, $P<0.0002$) level of detection. However, even the levels detected in chicken are negligible compared to the buffering capacity of hemoglobin (60.5 mmol/(L cell) at 340 g Hb/(L cell) **(2, 3)**).

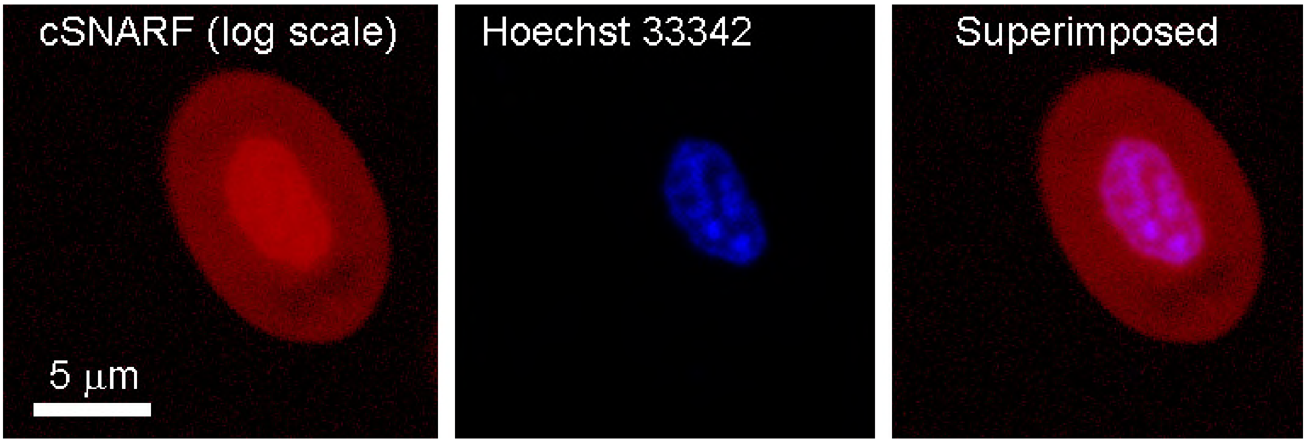

Fig. S5. *Nuclear regions in nucleated RBCs.* **(A)** cSNARF1 fluorescence (>580 nm) in chicken RBC (excitation: 555 nm), showing stronger signal in center of cell (fluorescence intensity is shown on a log scale). **(B)** Hoechst 33342 fluorescence (>440 nm) in nuclear region of chicken RBC (excitation: 405 nm). (C) Superimposition of cSNARF1 and Hoechst 33342 images shows that high cSNARF1 signal coincides with nucleoplasm.

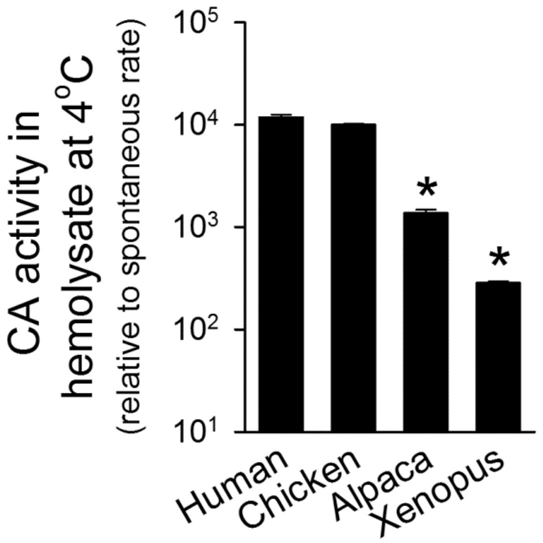

Fig. S6. *Carbonic anhydrase activity in hemolysates from different animal species.* Carbonic anhydrase (CA) activity was measured using the method shown in Fig. S3A (mean±SEM of at least 12 samples per animal). Hemolysates were diluted serially (at least four dilutions), and the measured hydration rate constants were back-extrapolated to 100% hemolysate in order to estimate native CA activity in cytoplasm (at 4°C). CA activity was defined as ratio of CA-catalyzed hydration rate to the spontaneous rate (measured in the presence of 200μM ATZ). * denotes significance ($P<10^{-4}$) relative to human data.

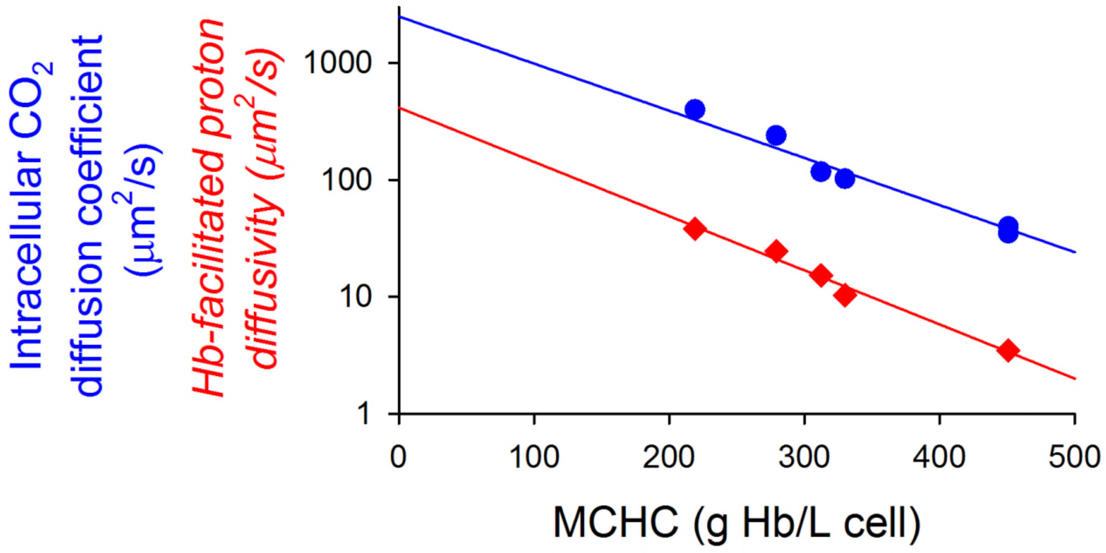

Fig. S7. *Diffusion in cytoplasm as a function of mean corpuscular hemoglobin concentration (MCHC).* Data for CO_2 diffusivity (blue) are from Fig. 3. Best fit constrained to 2500 $\mu m^2/s$ at MCHC=0 (pure water): D_{CO2}=2500×exp(−MCHC/107.7). Hemoglobin-facilitated H^+ diffusivity data (red) are equal to D_H^{app} measured in the absence of CO_2/HCO_3^- (Fig 1-2). Best fit: D_{Hb}=415×exp(−MCHC/93.7). For both correlations, $P<10^{-4}$.

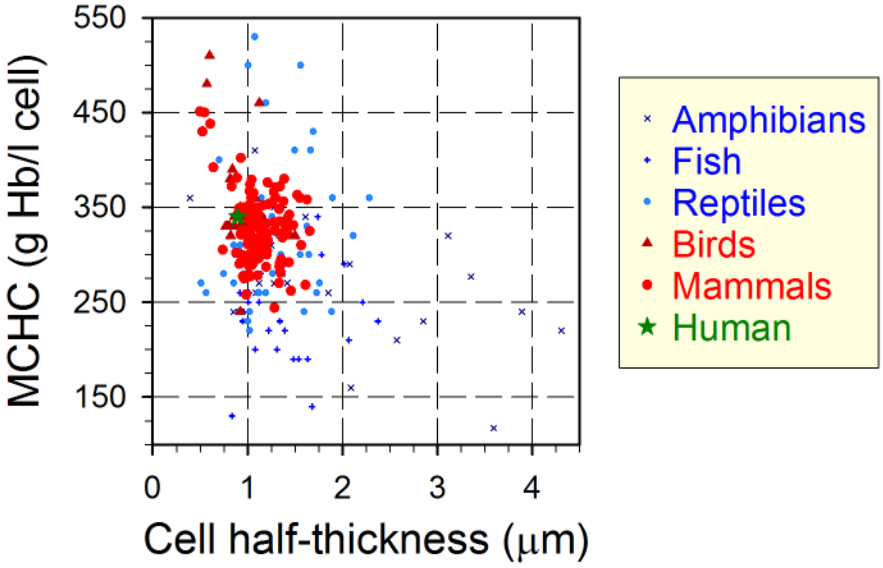

Fig. S8. *Relationship between RBC half-thickness and mean corpuscular hemoglobin concentration (MCHC).* Data from Table S2. Correlation between log(half-thickness) and MCHC: correlation coefficient (R), probability (P) and count (N) for entire data set: R=–0.282, P<0.0001, N=265; for mammals only: R=–0.365, P<0.0001, N=127; for birds only: R=–0.457, P<0.005, N=36.

Additional references

1. Swietach P, *et al.* (2008) Tumor-associated carbonic anhydrase 9 spatially coordinates intracellular pH in three-dimensional multicellular growths. *J Biol Chem* 283(29):20473-20483.
2. Cass A & Dalmark M (1973) Equilibrium dialysis of ions in nystatin-treated red cells. *Nat New Biol* 244(132):47-49.
3. Dalmark M (1975) Chloride and water distribution in human red cells. *J Physiol* 250(1):65-84.

Table S1: Hematological data for human (^{F}-female, ^{M}-male), alpaca, chicken and *Xenopus* blood. ^{C}-calculated from other data. ND-not determined.

Species	Source	Hematocrit (%)	[Hb] in blood (g/l)	MCV (fl)	MCHC (g/l)
Human	Merck Manual [1]	36-47%^{F}, 41-51%^{M}	120-160^{F}, 140-170^{M}	80-100	320-360
	Vajpayee 2011 [2]	40-45	125-165	80-100	320-360
	Khurana 2013 [3]	42-45	120-180	78-94	300-380
	**MEAN**	**43**	**147.5**	**88**	**340**
Alpaca	Merck Veterinary Manual [4]	29-39	128-177	20.9-28	441-454^{C}
	Tornquist 2015 [5]	25-45	113-190	22-29.5	422-452^{C}
	Dawson 2011 [6]	22-45	102-193	21-28	420-290
	Hadjuk 1992 [7]	30-42	144-188	26-31	448-490^{C}
	**MEAN**	**34.6**	**154**	**25.8**	**451**
Chicken	Bounous 2000 [8]	22-35	70-130	90-140	260-350
	Gulland 1990 [9]	30-49	102-151	104-135	302-362
	Ghergariu 2000 [10]	32-48	102-106	ND	220-319^{C}
	Gylstorff 1983 [11]	24-43	89-135	120-137	314-371^{C}
	**MEAN**	**35.4**	**110.6**	**121**	**312**
Xenopus	Jokumsen 1980 [12]	38.7	113.52	ND	265-293^{C}
	Wilson 2011 [13]	23.3-47	60.6-151.9	ND	193-323
	Wilson 2011 [13]	41.9-56.2	82.2-216.8	ND	190-405
	Wilson 2011 [13]	33.1-54.7	55.2-204.6	ND	167-378^{C}
	Horner 1983 [14]	ND	ND	250	ND
	**MEAN**	**41.5**	**116.4**	**250**	**270**

Additional references

1 Porter, R. S. in *Merck Manual Professional Edition* (Merck & Co, 2011).

2 Vajpayee N., Graham S. S. & S., B. in *Henry's Clinical Diagnosis and Management by Laboratory Methods* (ed Pincus MR McPherson RA) 509-535 (Elsevier Saunders, Philadelphia, 2011).

3 Khurana, I. *Textbook of Human Physiology for Dental Students*. 2nd edn, 72-73 (Elsevier 2013).

4 Kahn, C. M. in *Merck Veterinary Manual* (Merck & Co, 2010).

5 Tornquist, S. J. *Hematology of Camelids*, <http://www.alpacaresearch.org/library/library-viewer?article_id=2515> (

6 Dawson, D. R., DeFrancisco, R. J. & Stokol, T. Reference intervals for hematologic and coagulation tests in adult alpacas (Vicugna pacos). *Veterinary Clinical Pathology* **40**, 504-512 (2011).

7 Hajduk, P. Haematological reference values for alpacas *Australian Veterinary Journal* **69**, 89-90 (1992).

8 Bounous, D. I. & Stedman, N. L. in *Schalm´s Veterinary Hematology* (ed J.G. Zinkl and N.C. Jain B.F. Feldman) 1147-1154 (Lippincott, Williams and Wilkins, Philadelphia, 2000).

9 Gulland, F. M. D. & Hawkey, C. M. in *Veterinary Annual* 126-136 (1990).

10 Ghergariu, S., Pop, A., Kadar, L. & Spânu, M. in *Manual de laborator clinic veterinar* (All, Bucureşti, 2000).

11 Gylstorff, I. in *Handbuch der Geflügelphysiologie* (ed A. Mehner, W. Hartfiel) 280-393 (Jena, Veb Gustav Fischer Verlag, 1983).

12 Jokumsen, A. & Weber, R. E. Hemoglobin-Oxygen Binding-Properties in the Blood of Xenopus-Laevis, with Special Reference to the Influences of Estivation and of Temperature and Salinity Acclimation. *Journal of Experimental Biology* **86**, 19-37 (1980).

13 Wilson, S. *et al.* Serum clinical biochemical and hematologic reference ranges of laboratory-reared and wild-caught Xenopus laevis. *J Am Assoc Lab Anim Sci* **50**, 635-640 (2011).

14 Horner, H. A. & Macgregor, H. C. C-Value and Cell-Volume - Their Significance in the Evolution and Development of Amphibians. *Journal of Cell Science* **63**, 135-146 (1983).

Table S2: Mean corpuscular hemoglobin concentration (MCHC) and half-thickness of RBCs from different species of animal. Cell thickness was calculated as the ratio of mean corpuscular volume (MCV) to cross-sectional area (π × minor radius × major radius).

Species	MCHC (g/L)	MCV (fL)	Area (μm^2)	Half-thickness (μm)
**MAMMALS**				
Human [1,2]	340	90	50.2	0.896
Human [3]	328	91	40.808	1.115
Lama pacos (Alpaca) [4,5]	451	21.3	21.5	0.496
Lama pacos (Alpaca) [3]	402	29.8	16.046	0.929
Llama[4]	430	24	23.0	0.522
Lama glama [3]	392	28.3	22.2	0.638
Camel [4]	450	29.8	27.3	0.546
Camelus bactrianus [3]	438	27	22.3	0.606
Lemur catta [3]	295	77.5	28.6	1.353
Callithrix jaccus [3]	321	70.9	38.2	0.927
Aotus trivirgatus [3]	335	85.5	41.5	1.030
Cebus capucinus [3]	318	85.9	38.5	1.117
Cebus paella [3]	292	95.5	33.4	1.435
Cebus albifrons [3]	300	84.2	35.0	1.202
Cebus nigrivittatus [3]	292	88	41.4	1.063
Ateles paniscus [3]	325	78.8	38.5	1.024
Lagothrix lagothrica [3]	337	81.2	38.2	1.062
Macaca nemestrina [3]	300	79.3	38.9	1.019
Macaca fascicularis [3]	302	70	39.8	0.880
Macaca mulatta [3]	302	76.9	35.8	1.075
Macaca sylvana [3]	317	81.5	37.6	1.084
Macaca assamensis [3]	293	75.5	40.5	0.933
Macaca speciose [3]	295	76	38.5	0.988
Macaca maturus [3]	277	76.1	40.0	0.951
Macaca silenus [3]	275	78.7	40.9	0.962
Macaca sinica [3]	325	70.5	36.1	0.977
Cynopithecus niger [3]	258	79.3	40.4	0.983
Papio anubis [3]	328	89.4	39.1	1.142

Papio cynocephalus [3]	307	87.5	40.1	1.090
Papio papio [3]	302	91.4	39.7	1.152
Papio hamadryas [3]	312	91.1	38.8	1.174
Mandrillus sphinx [3]	288	87.8	43.2	1.016
Mandrillus leucophaeus [3]	300	88.5	41.3	1.072
Ceropithecus pygerethrus [3]	315	84	37.9	1.108
Ceropithecus sabaeus [3]	277	87.6	42.6	1.027
Ceropithecus mona [3]	302	77	32.8	1.175
Ceropithecus talapoin [3]	308	84.5	38.1	1.108
Ceropithecus sclateri [3]	310	86	41.0	1.048
Ceropithecus diana [3]	292	79	41.0	0.963
Erythrocebus patas [3]	296	83.9	37.6	1.116
Cercocebus torquatus [3]	330	90	42.4	1.061
Hylobates lar [3]	297	67.4	34.8	0.968
Hylobates hoolock [3]	278	82.2	40.7	1.010
Pan troglodytes [3]	297	87.8	40.7	1.079
Gorilla gorilla [3]	292	86.8	46.9	0.925
Pongo pygmaeus [3]	289	80.6	38.6	1.045
Canis lupus [3]	350	71.5	37.9	0.943
Canis latrans [3]	313	78.6	37.5	1.048
Canis familaris [3]	323	75.2	36.8	1.021
Canis familaris dingo [3]	332	77.5	35.4	1.093
Alopex lagopus [3]	299	59.6	31.3	0.953
Vulpes vulpes [3]	349	57	31.4	0.909
Urocyon cinereoargenteus [3]	279	58	28.6	1.013
Speothos venaticus [3]	287	85.2	35.7	1.195
Lycaon pictus [3]	322	68.2	24.8	1.375
Otocyon megalotis [3]	278	70.7	32.0	1.106
Tremarctos ornatus [3]	290	47.9	26.2	0.913
Selenarctos thibetanus [3]	335	75.7	35.8	1.058
Nasua nasua [3]	293	59.4	30.9	0.962
Procyon lotor [3]	299	60	27.1	1.105
Eira barbara [3]	309	63.5	28.7	1.105
Paradoxurus hermaphroditus [3]	326	39.5	14.1	1.399
Herpestes urva [3]	315	41.5	20.7	1.004

Felis caracal [3]	322	47.5	19.7	1.205
Felis leo [3]	323	52.5	22.4	1.173
Felis concolor [3]	365	48.2	22.7	1.061
Panthera tigris [3]	335	59.7	22.4	1.333
Panthera pardus [3]	334	48	18.8	1.279
Panthera onca [3]	325	55	21.2	1.296
Acinonyx jubatus [3]	318	58.5	21.4	1.367
Equus caballus [3]	353	51.9	21.4	1.213
Equus przewalski [3]	352	55.1	23.7	1.164
Equus hemionus [3]	325	68.1	20.6	1.655
Equus astinus [3]	380	61.8	22.3	1.386
Equus zebra [3]	343	46.1	20.7	1.111
Equus grevyi [3]	360	51	19.7	1.294
Tapirus terrestris [3]	262	50.5	17.3	1.456
Diceros bicornis [3]	379	69.2	33.4	1.040
Sus scrofa [3]	324	71	25.1	1.412
Potamochoerus porcus [3]	340	56.3	25.4	1.108
Tayassu tajacu [3]	270	61.4	23.1	1.331
Vicugna vicugna [3]	381	31.1	17.5	0.886
Camelus dromedarius [3]	322	39.9	20.9	0.955
Dama dama [3]	315	45.9	18.9	1.213
Cervus timorensis [3]	350	40	18.2	1.097
Cervus elephas [3]	333	43.9	15.8	1.387
Odocoileus virginianus [3]	338	46.5	16.4	1.418
Cervus canadensis [3]	372	38.9	23.4	0.831
Elaphurus davidianus [3]	367	50.1	24.5	1.021
Rangifer tarandus [3]	366	47.5	18.6	1.276
Giraffa camelopardalis [3]	348	39.4	14.8	1.332
Antilocapra americana [3]	311	41	20.5	1.000
Boselephas tragocamelus [3]	331	59	19.9	1.479
Tragelephas strepsiceros [3]	360	53.6	25.9	1.036
Bos taurus [3]	337	57.1	26.0	1.100
Taurotragus oryx [3]	353	51	24.9	1.025
Bos grunniens [3]	304	48.8	20.3	1.204
Bison bonasus [3]	287	59.7	22.4	1.333

Oryx leucoryx [3]	291	61.5	23.0	1.338
Connochaetes taurinus [3]	374	31.3	15.3	1.025
Damaliscus dorcas [3]	363	43.8	14.4	1.523
Antilope cervicapra [3]	355	44.6	17.7	1.259
Saiga tatarica [3]	281	58.9	21.9	1.346
Ovibos moschatus [3]	376	39.1	16.2	1.208
Capra ibex [3]	244	36.2	14.1	1.283
Capra hircus [3]	321	21.5	7.6	1.407
Ovis aries [3]	342	34.3	16.0	1.010
Ovis musimon [3]	345	31.8	14.9	1.070
Ovis tragelaphus [3]	268	29.2	9.1	1.609
Protemnodon rufogrisea [3]	341	90.3	42.6	1.059
Vombatus ursinus [3]	310	74	23.7	1.564
Oryctolagus cuniculus [3]	302	72.2	31.2	1.159
Mesocricetus auratus [3]	333	62.4	26.1	1.194
Rattus norvegicus [3]	322	61.8	33.4	0.926
Mus musculus [3]	312	52.9	23.0	1.151
Hydrochoerus hydrochaeris [3]	305	113.5	77.0	0.738
Cavia porcellus [3]	310	79.1	37.1	1.067
Myocaster coypus [3]	290	117	43.5	1.346
Elephas maximus [3]	325	125	65.9	0.949
Loxodonta africana [3]	350	128	64.4	0.993
Phocoenoides dalli [3]	371	96.3	37.4	1.288
Tursiops truncatus [3]	372	103	38.4	1.343
Globicephala scammoni [3]	358	117.9	36.3	1.624
Orcinus orca [M 3]	360	112.5	36.3	1.550
Lagenorhynchus obliquidens[M 3]	356	94.1	34.2	1.376
Inia geoffrensis [3]	342	107.6	37.4	1.440
Phoca vitulina [3]	314	107.6	44.4	1.212
**BIRDS**				
Gallus g.domesticus [6]	330	127	73.6	0.863
Pavo cristatus [6]	330	178	69.2	1.285
Gallus gallus bankiva [6]	340	137	59.6	1.149
White leghorn chicks [6]	240	119	64.6	0.921
Phasianus colchicus [6]	460	127	56.6	1.122

Numida meleagris [6]	330	140	91.1	0.769
Porphyrio porphyrio [6]	340	170	99.0	0.858
Burhinus oedicnemus [6]	300	167	71.0	1.176
Catharacta lonnbergi [6]	300	125	61.1	1.022
Larus marinus [6]	320	200	69.1	1.447
Larus argentatus [6]	320	201	67.0	1.499
Larus rudibundus [6]	360	141	65.5	1.076
Larus dominicanus [6]	320	107	65.3	0.820
Treron bicincta bicincta [6]	510	99	82.9	0.597
Columba livia [6]	330	144	89.0	0.809
Columba guinea [6]	390	125	74.6	0.838
Streptopelia senegalensis [6]	360	162	61.8	1.310
Caloenas nicobarica [6]	340	150	79.7	0.942
Otidiphaps nobilis [6]	340	159	80.5	0.987
Gourna cristata [6]	320	159	67.5	1.177
Gourna victoria [6]	330	167	88.2	0.947
Probosciger atterimus [6]	330	174	89.9	0.968
Cacatua alba [6]	340	151	81.2	0.929
Cacatua goffini [6]	350	150	81.7	0.918
Calyptorhynchus funereus [6]	340	166	88.3	0.941
Eos bornea [6]	330	156	85.2	0.915
Psittacula krameri borealis [6]	480	92	80.6	0.571
Psittacula erithacus [6]	330	145	73.6	0.985
Cyanoliseus patagonus [6]	320	135	66.8	1.010
Ara ararauna [6]	330	141	62.2	1.134
Ara chloroptera [6]	300	145	77.8	0.932
Ara macao [6]	340	152	88.4	0.860
Ara militaris [6]	340	137	76.3	0.898
Apus apus [6]	330	116	73.7	0.787
Tyto alba [6]	340	170	88.3	0.962
Bubo africanus [6]	380	160	98.1	0.815
**AMPHIBIANS**				
Tree frog (male) [7]	117.4	851.31	118.5	3.591
Northern Banded Newt [M 8]	277.2	3128.07	466.3	3.354
Cryptobranchus aileganimsisa [9]	230	5973	1047.7	2.851

Crypohchus alfeganiends bishop [9]	210	4323	840.0	2.573
Nectusm aculosud [9]	220	10070	1168.8	4.308
Amphiuma mean [9]	240	13857	1781.0	3.890
Dicampbdon ensatusl [9]	160	4938	1182.2	2.088
Triturus cristatus carnifIe [9]	320	2820	453.1	3.112
Plethodon glutinmus [9]	290	2200	529.6	2.077
Desmognathus quadramaculatus [9]	260	1552	419.5	1.850
Rana catesbeianal [9]	270	671	299.8	1.119
Rana esculent [9]	410	459	212.9	1.078
Rana tigern (male) [9]	360	186	237.4	0.392
Rana cyanophlyctis (male) [9]	240	219	128.7	0.851
Rana pipiend [9]	270	773	302.7	1.277
Bufo melanostictus (male) [9]	340	465	144.4	1.610
Bufo spinulosus, low altitude [9]	270	544	191.7	1.419
Bufo spinulosus, high altitude [9]	280	451	164.9	1.368
Telmatobius marmoratus [9]	310	448	179.6	1.247
Telmatobius halli [9]	290	390	145.3	1.342
Telmatobius pefauri [9]	320	338	139.0	1.216
Telmatobius peruvianus [9]	350	446	174.8	1.275
Boulengerula taitanus [9]	260	588	270.6	1.086
**FISH**				
Myxine glutinosa [10]	210	1530	370.2	2.066
Petromyzon marinus [10]	250	710	160.5	2.211
Mustelus canis [10]	200	541	206.9	1.307
Torpedo ocellaata [10]	130	1039	621.7	0.836
Raja eglanteria [10]	190	823	267.9	1.536
Raja laevis [10]	190	1274	390.1	1.633
Acipenser sturio [10]	230	506	106.6	2.373
Salmo gairdneri [10]	190	380	128.3	1.482
Esox lucius [10]	280	169	74.3	1.138
Carassius auratus [10]	260	178	87.7	1.015
Cyprinus carpio [10]	340	373	107.3	1.738
lctiobus bubalus [10]	260	257	108.1	1.189
lctalurus nebulosus [10]	250	228	101.8	1.120
Anguilla rostrata [10]	240	156	81.6	0.955

Gadus morhua [10]	200	186	86.2	1.079
Micropterus salmoides [10]	220	166	59.7	1.391
Trernatomus borchgrevinki [10]	140	319	95.0	1.679
Scomber scornbrus [10]	260	147	80.1	0.917
Thunnus alalunga [10]	290	188	46.6	2.016
Katsuwonus pelamis [10]	300	143	40.2	1.779
Pnonotus carolinus [10]	300	96	50.0	0.960
Pnonotus evolans (strigatus) [10]	280	130	59.6	1.091
Myoxocephalus Scorpius [10]	220	214	87.6	1.221
Lirnanda ferruginea [10]	230	118	62.3	0.948
Opsanus tau [10]	230	404	150.9	1.339
Lophius piscatorius [10]	250	204	101.4	1.006
Helostoma temmincki [10]	300	75	37.3	1.006
Channa punctatus [10]	260	154	78.5	0.981
**REPTILES**				
Macroclemys temminckii [11]	290	552	282.6	0.977
Chelydra serpentina serpentina [11]	590	1364	256.8	2.656
Terrapene carolina carolina [11]	410	677	203.8	1.661
Chrysemys picta marginata [11]	410	696	232.9	1.494
Pseudemys scripta troostii [11]	320	706	167.3	2.111
Pseudemys elegans [11]	240	427	209.2	1.021
Mauremys caspica leprosa (summer) [11]	460	417	184.9	1.128
Mauremys caspica leprosa (autumn) [11]	460	451	189.4	1.191
Emys orbicularis (male) [11]	310	478	259.7	0.920
Testudo graeca [11]	300	416	154.0	1.351
Testudo hermanni	260	515	149.2	1.726
Gopherus polyphemus [11]	280	424	168.0	1.262
Geochelone gigantea (male) [11]	240	544	298.3	0.912
Trionyx gangeticus (male) [11]	380	266	158.9	0.837
Chelonia mydas [11]	240	1320	140.4	4.700
Caretta caretta [11]	270	546	536.9	0.508
Crocodylus niloticus [11]	320	307	115.9	1.325
Crocodylus porosus [11]	320	225	94.0	1.197
Crocodylus johnsoni [11]	340	236	94.0	1.256

Alligator mississippiensis [11]	360	516	136.3	1.893
Caiman latirostris (male) [11]	500	396	127.1	1.558
Caiman crocodilus yacre (male) [11]	430	430	127.1	1.691
Hemidactylus flaviviridis (male) [11]	400	203	145.1	0.700
Crotaphytus collaris (male) [11]	290	336	120.2	1.398
Iguana iguana (male) [11]	220	192	94.0	1.021
Tropiduras torquatus [11]	240	334	104.8	1.593
Physignathus lesueurii [11]	270	385	225.5	0.854
Agama agama (male) [11]	230	389	194.4	1.001
Chamaeleo dilepis [11]	300	300	91.2	1.644
Leiolopisma smithi [11]	300	464	149.5	1.552
Leiolopisma zelandica[11]	530	301	140.3	1.072
Tiliqua rugosa [11]	260	277	125.0	1.108
Tiliqua scincoides [11]	330	300	125.0	1.200
Cordylus vittifer [11]	240	415	110.1	1.885
Podarcis sicula sicula (spring) [11]	260	118	105.0	0.562
Psammodromus algirus (male) [11]	260	189	84.5	1.118
Varanus bengalensis (male) [11]	330	237	125.3	0.946
Varanus niloticus [11]	300	359	203.9	0.880
Varanus monitor (male) [11]	360	340	148.6	1.144
Acrochordus granulatus [11]	330	932	287.0	1.624
Natrix piscator piscator [11]	340	246	142.0	0.866
Natrix natrix natrix (male) [11]	280	191	128.0	0.746
Dispholidus typus (male) [11]	270	196	55.8	1.758
Coluber ventromaculatus (male) [11]	500	239	119.1	1.004
Atretium schistosum (male) [11]	300	271	156.7	0.865
Pseudonaja nuchalis [11]	290	314	156.6	1.002
Pseudechis porphyriacus [11]	280	289	150.1	0.963
Notechis scutatus [11]	340	369	158.9	1.161
Austrelaps superbus [11]	310	258	151.2	0.853
Naja haje haje [11]	360	273	59.8	2.281
Crotalus cerastes [11]	260	412	173.3	1.189

Additional references

1 Vajpayee N, G. S., Bem S. in *Henry's Clinical Diagnosis and Management by Laboratory Methods* (ed Pincus MR McPherson RA) 509-535 (Elsevier Saunders, Philadelphia, 2011).

2 Smith, J. E., Mohandas, N. & Shohet, S. B. Variability in Erythrocyte Deformability among Various Mammals. *American Journal of Physiology* **236**, H725-H730 (1979).

3 Hawkey, C. M. *Comparative mammalian haematology : cellular components and blood coagulation of captive wild animals*. (Heinemann Medical, 1975).

4 Yamaguchi, K., Jurgens, K. D., Bartels, H. & Piiper, J. Oxygen-Transfer Properties and Dimensions of Red-Blood-Cells in High-Altitude Camelids, Dromedary Camel and Goat. *Journal of Comparative Physiology B-Biochemical Systemic and Environmental Physiology* **157**, 1-9, doi:Doi 10.1007/Bf00702722 (1987).

5 Dawson DR, D. R., Stokol T. . Reference intervals for hematologic and coagulation tests in adult alpacas (Vicugna pacos). *Veterinary Clinical Pathology* **40**, 504-512 (2011).

6 Glomski, C. A. & Pica, A. *The avian erythrocyte : its phylogenetic odyssey*. (Science Publishers, 2011).

7 Das, M. & Mahapatra, P. K. Hematology of Wild Caught Dubois's Tree Frog Polypedates teraiensis, Dubois, 1986 (Anura: Rhacophoridae). *Scientific World Journal*, doi:Artn 491415
10.1155/2014/491415 (2014).

8 Murat Tosunoglu, C. V. T., Kurtulus Olgun, Nurhayat Özdemir, Çigdem Gül. Hematology of the Northern Banded Newt, *Ommatotriton ophryticus* (Amphibia: Urodela), from North Anatolia. *Russian Journal of Herpetology* **18**, 59-64 (2011).

9 Glomski, C. A., Tamburlin, J., Hard, R. & Chainani, M. The phylogenetic odyssey of the erythrocyte .4. The amphibians. *Histology and Histopathology* **12**, 147-170 (1997).

10 Glomski, C. A., Tamburlin, J. & Chainani, M. The Phylogenetic Odyssey of the Erythrocyte .3. Fish, the Lower Vertebrate Experience. *Histology and Histopathology* **7**, 501-528 (1992).

11 Glomski, C. A. & Pica, A. *Erythrocytes of the poikilotherms : a phylogenetic odyssey*. (Foxwell & Davies, 1992).
